# Identification of Antioxidant Metabolites from Five Plants (*Calophyllum inophyllum*, *Gardenia taitensis*, *Curcuma longa*, *Cordia subcordata*, *Ficus prolixa*) of the Polynesian Pharmacopoeia and Cosmetopoeia for Skin Care

**DOI:** 10.3390/antiox12101870

**Published:** 2023-10-16

**Authors:** Marion Chambon, Raimana Ho, Beatrice Baghdikian, Gaëtan Herbette, Sok-Siya Bun-Llopet, Elnur Garayev, Phila Raharivelomanana

**Affiliations:** 1 UMR 214 Ecosystèmes Insulaires Océaniens, Université de Polynésie Française, IFREMER, ILM, IRD, BP 6570, Tahiti, F-98702 Faaa, French Polynesia; marion.chambon@doctorant.upf.pf (M.C.); raimana.ho@upf.pf (R.H.); 2 Aix Marseille Univ, CNRS 7263, IRD 237, Avignon Université, IMBE, 27 Blvd Jean Moulin, Service of Pharmacognosy, Faculty of Pharmacy, 13385 Marseille, France; beatrice.baghdikian@univ-amu.fr (B.B.); sok-siya.bun@univ-amu.fr (S.-S.B.-L.); elnur.garayev@univ-amu.fr (E.G.); 3 Aix Marseille Université, CNRS, Centrale Méditerranée, FSCM, Spectropole, Service 511, Campus Saint-Jérôme, 13397 Marseille, France; gaetan.herbette@univ-amu.fr

**Keywords:** *Calophyllum inophyllum*, *Gardenia taitensis*, *Curcuma longa*, *Cordia subcordata*, *Ficus prolixa*, French Polynesia, cosmetopoeia, pharmacopeia, LC-MS, antioxidant

## Abstract

Oxidative stress contributes to impairment of skin health, the wound healing process, and pathologies such as psoriasis or skin cancer. Five Polynesian medicinal plants, among the most traditionally used for skin care (pimples, wounds, burns, dermatoses) are studied herein for their antioxidant properties: *Calophyllum inophyllum*, *Gardenia taitensis*, *Curcuma longa*, *Cordia subcordata*, and *Ficus prolixa*. Plant extracts were submitted to in vitro bioassays related to antioxidant properties and their bioactive constituents were identified by a metabolomic analytical approach. High performance liquid chromatography with tandem mass spectrometry (HPLC-MS/MS) analysis was performed leading to the characterization of 61 metabolites. Compounds annotated for *F. prolixa* and *C. subcordata* extracts were reported for the first time. Antioxidant properties were evaluated by total phenolic content (TPC), free radical scavenging DPPH (1,1-diphenyl-2-picryl-hydrazyl), and Ferric Reducing Antioxidant Power activity (FRAP) assays. *F. prolixa* extract was the most active one and showed antioxidant intracellular activity on keratinocytes by Anti Oxydant Power 1 assay. Online HPLC-DPPH allowed the identification of phenolic bioactive compounds such as quercetin-*O*-rhamnoside, rosmarinic acid, chlorogenic acid, procyanidins, epicatechin, 5-*O*-caffeoylshikimic acid, and curcumin as being responsible for the scavenging properties of these plant extracts. These results highlight the potential of *F. prolixa* aerial roots as a source of antioxidants for skin care applications.

## 1. Introduction

The skin is the largest organ of the human body. On the outer layer (epidermis), the skin is composed of keratinocytes, and on the second layer (dermis), it is composed of fibroblasts. It establishes a protective barrier between the organism and the external environment, and against UV radiation, air pollution, chemical components (drug, cosmetics, etc.), and different pathogen threats [1]. In response to these external aggressions, keratinocytes produce reactive oxygen species (ROS) aiming to activate cell proliferation and survival. However, on the other hand, ROS production may damage DNA, collagen structures, and lipid membranes [2]. This oxidative stress is regulated by enzymatic and non-enzymatic antioxidants creating a balance between the ROS production system and the antioxidant system [2]. In case of damaging threats or pathologies such as diabetes, these systems and their natural balance are disturbed, contributing to the generation of impairment in the wound healing process as well as inducing acne, psoriasis, or skin cancer [2,3]. Different therapeutic strategies are possible to help avoid such cutaneous disorders. The most commonly used strategy is to reduce the level of ROS by the use of an additional antioxidant administered orally or topically.

Skin diseases pose an issue in French Polynesia where they are particularly enhanced by the occurrence of higher rates of diabetes [4] and sun exposure. On the other side, the geographical isolation of islanders living far away from city hospitals may reduce their access to healthcare and pharmaceuticals. French Polynesia is a territory with rich plant biodiversity that is used in well-known traditional medicines and practices of pharmacopeia as well as cosmetopeia [5]. Plants have been used for centuries in health and skin care and they can provide a natural source of antioxidant molecules, including mainly glutathione, ascorbic acid, carotenoids, tocopherols, and phenolic compounds (flavonoids, coumarins, xanthones, phenolic acids, tannins) [6].

To address this issue, Polynesian plants used for wound healing and anti-inflammatory and antioxidant properties were studied. The selection of the herein-investigated plants had been achieved following four main criteria. First of all, the selected plants must have ethnobotanical uses in Polynesian traditional medicine (*Raau* Tahiti) for burns, wounds, dermatoses, pimples, itch, rashes, and other skin treatments. Secondly, their bio-ecological status should be considered to exclude protected, endangered, and rare plants (from the UICN list) to keep only abundant and renewable ones. Thirdly, the biogeographic status had been also used to exclude modern introduced plants (settled after European arrival in Polynesia), as they do not have long-term Polynesian ancestral use, so we are keeping indigenous and Polynesian introduced plants brought by Polynesian first settlements and immigrants in the Oceania region. Then, the importance of the selected plant phytochemical data from the literature was also considered. This network led to a short list of five selected plants, used for skin care, and some of them were known to be incorporated into “*Monoi*” which is a local coconut oil preparation that includes macerated plants. The plants are are among the most commonly used in French Polynesia in traditional medicine and skin care [5]:

*Calophyllum inophyllum* L. (Calophyllaceae) is an evergreen tree called “*Tamanu*” in Tahiti. *C. inophyllum* nuts are sun-dried for 2–3 months and cold-pressed to yield viscous oil ubiquitously used in *Raau* Tahiti to treat burns, sunburn, infected wounds, eczemas, and to heal many skin problems [5,7]. *C. inophyllum* leaves are also used in traditional recipes to cure dermatosis, itch, or inflammation [7,8].

Flowers of *Gardenia taitensis* DC trees (Rubiaceae) called locally “*Tiare*” are emblematic and flagship flowers of Tahiti island. They are used as a major cosmetic ingredient, being commonly macerated in coconut oil yielding *Monoi* which is applied for daily skin and hair care. They are also mentioned in numerous recipes to treat infected wounds, dermatosis, contusion, skin abscesses, and cutaneous allergies [5,8,9].

*Curcuma longa* L. (Zingiberaceae) named “*Rea*” is an herbaceous plant with tuberous rhizomes widely used in food, cosmetics, medicine, and as a yellow dye. In *Raau* Tahiti, they are incorporated into wound healing or skin abscess treatment [8]. They are also added in *Monoi*.

The two last selected plants for this study are two indigenous trees: *Cordia subcordata* Lam. (Boraginaceae) called “*Tou*”, a medium-sized evergreen tree growing in coastal areas, and *Ficus prolixa* G. Forst (Moraceae) named “*Ora*”, a sacred banyan with a complex trunk formed of anastomosed filiform aerial roots. Green leaves of *C. subcordata* and aerial roots of *F. prolixa* are both traditionally used to treat cutaneous allergy, dermatosis, inflammation, and wounds [5,7,8,9]. The studied plants are presented in Table S1 of Supplementary data.

The present study focuses on the antioxidant capacity of these five Polynesian plants aiming to determine the antioxidant properties of their extracts through in vitro bioassays and to identify their bioactive components by a metabolomics analytical approach.

For this purpose, ultrasound-assisted extractions (UAE), a well-known eco-friendly process saving extraction time and solvent quantity use, were performed on plant materials to obtain crude extracts constituting the studied samples. Then, antioxidant properties were evaluated in samples by, respectively, analysis of total phenolic content (TPC), scavenging free radical ability (DPPH assay), metal-reducing activity (FRAP assay), and cellular-based Antioxidant Power 1 (AOP1) assay. The On-Line-HPLC-DPPH method also was used to assign bioactive radical scavenging constituents. Finally, LC-MS/MS data were used to create molecular networking through MZmine 3 and GNPS to characterize plant extracts metabolite contents.

## 2. Materials and Methods

### 2.1. Plant Materials

#### 2.1.1. Collection and Preparation

The five selected plants are some of the most used plants in Polynesian traditional medicine, especially for skin application. All plant parts were collected between January and May 2022 in French Polynesia: leaves of *C. inophyllum* L. (GPS coordinates: −17.631237; −149.614159), leaves of *C. subcordata* Lam. (−17.576955; −149.610608), nuts of *C. inophyllum* L. (−17.4822; −140.4539), aerial roots of *F. prolixa* G. Forst (−17.678171; −149.587278), and flowers of *G. taitensis* DC (−17.736932; −149.282326). *C. longa* L. rhizomes were bought from local markets. Plants were identified by the botanist J-F. Butaud and voucher specimens were deposited at the herbarium of French Polynesia (PAP). They were then oven-dried at 40 °C, except *C. inophyllum* nuts, which had been sundried for 8 weeks. So dried, plant parts were ground into a powder of 1–3 mm using an IKA MF 10 basic grinder.

#### 2.1.2. Ultrasound-Assisted Extraction

Ultrasound-assisted extraction (UAE) was performed using the PEX 1N-cs (24 kHz, 150 W, Reus France) with the homogenizer HS-50A Witeg. A constant temperature of 39 °C was maintained by the refrigerant system CF30 Julabo. The plant powder (80 g) of each sample was extracted for the first time in 550 mL of ethanol/water (70:30; *v*/*v*) for 30 min. This first extracted sample was then filtered and submitted to a second extraction for 30 min in renewed solvent. Solvent was removed from extracts by vacuum rotary evaporation and lyophilisation operations (Martin Christ Beta 2-8 LSCbasic, Osterode am Harz, Germany) to yield crude extract as the starting materials from which were performed all further analytical investigations.

#### 2.1.3. Liquid/Liquid Extraction

Liquid/Liquid extraction was performed by dissolving 1 g of crude extract in 20 mL of water. The obtained solution was submitted to a fractionation using the SPE (LLE/SLE) column CHROMABOND XTR, 70 mL/14,500 mg (Macherey-Nagel 730507, lot 2315.232, Dueren, Germany), and the crude extract constituents were eluted, respectively, with stepwise gradient solvents of 100 mL of hexane, then dichloromethane, ethyl acetate, and finally n-butanol. The solvents were removed from fractions by a vacuum rotary evaporation and lyophilisation operations (Cryotec, Saint-Gély-du-Fesc, France).

### 2.2. Total Phenolic Content, Radical Scavenging, and Antioxidant Activity

#### 2.2.1. Total Phenolic Content (TPC)

The total phenolic content was determined following the Folin–Ciocalteu colorimetric method adapted from El Hosry et al. [10]. The crude extracts were prepared at a concentration of 3 mg/mL in ethanol/water (50:50, *v*/*v*). In a 100 mL volumetric flask, 5 mL of the prepared solutions were mixed with 1 mL of Folin–Ciocalteu reagent (Sigma Aldrich, lot BCBP2077V, Saint-Louis, MO, USA), 4 mL of Na_2_CO_3_ 7.5% (m/v) (Honeywell Fluka Biochemika, 347579/1 596 lot.71345, Charlotte, NC, USA), and completed with distilled water. Samples were incubated for 2 h 30 in the dark at 30 °C in an oven. Absorbance of the solutions was measured at 760 nm using a UV/Vis spectrophotometer (Thermo Scientific Genesys 10S, Waltham, MA, USA). TPC was expressed as mg of gallic acid equivalent (GAE) per g of extracts.

#### 2.2.2. Determination of DPPH Radical Scavenging Activity

Free radical DPPH (1,1-diphenyl-2-picryl-hydrazyl) scavenging capacity assay was performed according to Blois et al. [11] and adapted for a 96-well plate (Sterilin Ltd., Newport, UK). A fresh DPPH (Sigma Aldrich, D9132-5G lot STBD2362V) solution was prepared everyday by dissolving the reagent (4 mg) in methanol (100 mL), kept at room temperature in the dark for 3 h before use. Then, positive control and samples were diluted with methanol to obtain different final concentrations in the wells: 3–15 µg/mL for ascorbic acid (Sigma Aldrich lot SLBB4446) and 5–100 µg/mL for crude extracts except for *F. prolixa* which needed lower concentrations, 1.25–50 µg/mL. The plate plan was realized according to Breaud et al. [12] and was composed of a blank row (250 µL of MeOH), a negative control row (50 µL of MeOH with 200 µL of DPPH solution), a positive control, and samples at different concentrations in triplicate (50 µL of positive control or sample with 200 µL of DPPH solution). The 96-well plate was placed in the microplate spectrophotometer (BioTek EON, Providence, RI, USA) and was incubated for 1 h at 30 °C. Absorbance was then read at 517 nm. The percentage of DPPH-H was calculated using the following formula (where Abs stands for absorbance):

% of DPPH-H = [(Abs_control_ − Abs_sample_)/Abs_control_] × 100

Then, the concentration providing 50% efficiency (EC_50_) in µg/mL was calculated using the equation of the polynomial curve expressing the percentage of DPPH-H in relation to the concentration. Statistical analysis was performed by ordinary one-way ANOVA test followed by Dunnett’s multiple comparisons tests.

#### 2.2.3. Determination of Ferric Reducing Antioxidant Power

The ferric reducing antioxidant power (FRAP) was determined using a modified version of the FRAP assay described by Benzie and Strain [13]. The assay was performed in a 96-well plate (Sterilin Ltd., Newport, UK). First, the FRAP solution reagent was prepared by mixing one volume of 10 mM TPTZ (2,4,6-tri(2-pyridyl)-s-triazine) reagent (Honeywell Fluka lot BCBK6346V, Charlotte, NC, USA) (in solution with 40 mM hydrochloric acid), one volume of 20 mM Iron(III) chloride hexahydrate (Honeywell Riedel-de-Haën lot 02550), and ten volumes of 300 mM sodium acetate buffer (pH 3.6). Then, positive control and samples were diluted with distilled water to obtain different final concentrations in the wells: 0.5–5 µg/mL for ascorbic acid (Sigma Aldrich lot SLBB4446) and 2.5–20 µg/mL of crude extracts. The plate was composed of a blank row (50 µL of distilled water with 200 µL of FRAP solution), a positive control, and samples at different concentrations in triplicate (50 µL of positive control or sample with 200 µL of FRAP solution). The 96-well plate was placed in the microplate spectrophotometer (BioTek EON, Providence, RI, USA) and incubated for 30 min at 40 °C. The absorbance was read at 593 nm. The obtained data were calculated and expressed as the FRAP value in mmol Fe^2+^/g. Statistical analysis was performed by ordinary one-way ANOVA test followed by Dunnett’s multiple comparisons tests.

#### 2.2.4. Antioxidant Power 1 (AOP1) Assay on Keratinocytes

This assay used a Light-Up Cell System (LUCS) patented approach based on the production of cellular radical species following the addition in the culture medium of a photo-inducible fluorescent nucleic acid biosensor [14]. The effect of light application in the presence of the cellular biosensor triggers the production of singlet oxygen which, in turn, causes the production of ROS in a biochemical cascade linked to an increase in emitted fluorescence. The effect is measured by a delay in the kinetic evolution of fluorescence emission. This method allows the evaluation of intracellular antioxidant activity.

A sample of crude extract of *F. prolixa* was kept at 4 °C and solubilized at a final concentration of 50 mg/mL in DMEM culture medium. A centrifugation at 8700 rpm for 10 min was added. Experiments were carried out with the supernatants. Human HaCaT cells, from the American Type Cell Collection (catalog number CRL-2404), were seeded in 96-well plates at a density of 40,000 cells/well in DMEM supplemented with Fetal Calf Serum (FCS) and kept in the incubator for 24 h at 37 °C/5% CO_2_. After the 1 h incubation with the fluorescent biosensor, cells were then incubated in the presence of samples (8 concentrations obtained by serial log2 dilutions) for 1 h at 37 °C/5% CO_2_. Experiments were realized in DMEM without FCS. At least two independent experiments were realized, each on triplicate wells.

Fluorescence was measured (RFU at 535 nm) according to a recurrent 480 nm LED application procedure (20 iterations) of the whole 96-well plate. Kinetic profiles were recorded. The sample monograph presents raw RFU values recorded during the kinetic analysis for each tested concentration and the corresponding normalized values. Antioxidant cell index (AOP index) is calculated from normalized kinetic profiles according to the formula:

AOP index (%) = 100 − 100 (_0_∫ ^20^ RFU_sample_/_0_∫ ^20^ RFU_control_)

By compiling AOP indices according to logarithm (10) of the sample concentration, dose–response curves were obtained and submitted to a sigmoid fit according to the following formula (SC = sample concentration and HS = Hill slope):

AOP index = AOP index_min_ + (AOP index_max_ − AOP index_min_)/(1 + 10^(Log(EC50-SC)*HS)^)

EC_50_ (50% efficacy concentration), EC_10_, and EC_90_ are then evaluated.

#### 2.2.5. Online RP-HPLC-DPPH

All method details and the instrumental setup were described by Breaud et al. [12]. This method allowed for the identification of radical scavenging compounds in crude extracts. Compounds were first separated with an HPLC Agilent 1260 system and detected with a DAD UV detector (DAD G7117C) at 325, 280, 254, and 210 nm. Then, compounds reacted with the DPPH solution (80 µg/mL) in a coil (25 m × 0.25 mm, corresponding to a contact time of 1 min 14 s) delivered by another HPLC pump (quaternary pump G1311A) at 60 °C with a flow rate of 0.2 mL/min. The final reaction solution is detected by a DAD UV-Vis detector (DAD G1315B) at 515 nm. The Agilent Zorbax Eclipse Plus C18 column (2.1 × 100 mm, 1.8 µm) at 43 °C was used for chromatographic separation. The mobile phase was composed of ultrapure water (A) and acetonitrile (B) (Carlo Erba, Milan, Italy), both acidified with 0.1% formic acid (Carlo Erba, Italy), and the following gradient was applied: isocratic hold 2 min at 5% B, 5–50% B over 2–17 min, 50–100% B over 17–27 min, then isocratic hold 2 min at 100% B (27–29 min). This was then followed by a decrease to 5% B for the column’s equilibration. Crude extracts were prepared at a concentration of 10 mg/mL. The injection volume was 1 µL and the flow rate was 0.2 mL/min. For annotation peak, the same gradient and column were used for both Online RP-HPLC-DPPH and LC-MS/MS analysis.

### 2.3. UHPLC-MS/MS Analysis

The high-performance liquid chromatography analyses were performed on a Dionex Ultimate 3000 (Thermo Scientific^®^) system equipped with a Photo Diode Array detector and coupled to a High-Resolution Mass Spectrometer (Bruker Impact II QToF) equipped with an electrospray ionization source. The sample solutions were prepared by solubilizing 1 mg of dry crude extract in 1 mL of ethanol/water (70:30, *v*/*v*). The chromatographic separation was carried on an Agilent Zorbax Eclipse Plus C18 column (2.1 × 100 mm, 1.8 µm) at 43 °C. The mobile phase was composed of ultrapure water (A) and acetonitrile (B), both acidified with 0.1% formic acid, and the following gradient was applied: isocratic hold 2 min at 5% B, 5–50% B over 2–17 min, 50–100% B over 17–27 min, then isocratic hold 2 min at 100% B (27–29 min). For the column’s equilibration, this was then followed by a decrease to 5% B in 1 min and held for 3 min. The injection volume was 1 µL and the flow rate was 0.8 mL/min. A sodium formate acetate calibration solution was injected at the beginning of each run as a calibration. Mass spectra were acquired using both positive and negative modes in a mass range of m/z 50 to 1200 and the following parameters were applied for the quadrupole time-of-flight (Q-TOF): end plate offset at 500 V; nebulizer N_2_ pressure at 3.5 Bar; dry N_2_ flow at 12 L/min; drying temperature at 200 °C; acquisition rate at 4 Hz; capillary voltage at 3500 V for positive mode and 3000 V for negative mode; stepped collision energy 20–40 eV.

### 2.4. Molecular Network

Raw data from UHPLC-MS/MS analysis were calibrated with Bruker Compass Data Analysis 5.0 SR1 (64-bit) and converted into mzXML with GNPS Vendor Conversion master. Data were then imported to MZmine 3.3.0 and processed with the following workflow: mass detection, ADAP chromatogram builder, chromatogram resolving (local minimum resolver), 13C isotope filter, alignment (join aligner), assign MS2 to features, feature list rows filter and blank subtraction. Parameters are described in Table S2 of Supplementary Data. Molecular networks were generated by using GNPS [15]. Annotation was facilitated with Sirius 5.6.3 [16].

## 3. Results and Discussion

### 3.1. Extraction

The ultrasound-assisted extraction (UAE) method was performed using 80 g of plant powder in 550 mL of ethanol/water (70:30, *v*/*v*). This extraction technique provided satisfying yields ranging from 16.5 to 33.1% (m/m) depending on the studied plant (Table 1). Ultrasound creates cavitation bubbles in the plant tissue that keep growing until they finally collapse. This process destroys plant material structure by breaking the cell wall and thus releasing molecules into the solvent to ease a quicker and better extraction process [17]. Moreover, ethanol can be considered as a biobased solvent for a greener extraction approach.

The UAE method allowed for reduced extraction duration compared to conventional methods, therefore saving time and energy. Indeed, Hughes, et al. [18] applied a 12 h maceration to *C. inophyllum* nuts that provided, respectively, extract yields of 0.76% with water solvent, 10.32% with ethanol/water (50:50, *v*/*v*), and 11.98% with ethanol, whereas one hour of UAE process led to a higher yield of 33.1% with ethanol/water (70:30, *v*/*v*). Likewise, the herein UAE method gave a yield of 21.5% for *C. inophyllum* leaves, whereas a percolation method at room temperature for 24 h with 95% ethanol led to a 12% extract yield [19]. In the same manner, *C. longa* extract yield was higher with the UAE process (24.6%) than with the Soxhlet extraction method (8.9%) described by Patil et al. using ethanol 99% for 12 h at 60 °C [20]. For these last two examples (*C. inophyllum* and *C. longa*), the UAE method allowed for the obtaining of higher extract yields within a shorter extraction time.

The choice of taking abundant plants and renewable parts in Polynesia for the present study aimed to promote environment preservation.

### 3.2. Total Phenolic Content (TPC)

Natural antioxidants found in plants are mainly composed of phenolic compounds such as flavonoids, coumarins, xanthones, phenolic acids, tannins, etc. Thus, the total phenolic content of crude extracts was assessed following the Folin–Ciocalteu method [10]. The results were expressed in gallic acid equivalent (GAE) and presented in Table 1.

For the *G. taitensis* flowers extract, a phenolic amount of 75 mg GAE/g was found. *C. inophyllum* nuts showed a TPC of 71 mg GAE/g, probably due to the high proportion of lipids in this oily extract inducing a dilution effect. Cassien et al. also obtained low TPC in a similar extract (14 mg GAE/g) [21]. *C. inophyllum* leaves extract showed a higher rate than nuts, with a TPC of 143 mg GAE/g, which was in good agreement with Hapsari et al. who reported a quite similar value of TPC for ethanolic extract (124.89 mg GAE/g) [22]. Indeed, *C. inophyllum* leaves are known to contain coumarins, xanthones, and flavonoids compounds, which may contribute to this TPC value [23]. A TPC value of 140 mg GAE/g was obtained for *C. longa* rhizomes, due to their phenolic content being constituted mainly of curcuminoid compounds. A previous study conducted by Singh et al. reported a TPC of 112.50 mg GAE/g for a turmeric extract obtained by UAE using ethanol solvent, thus showing an almost similar TPC value to our studied *C. longa* rhizomes extract [24]. *C. subcordata* extract had a TPC value of 139 mg GAE/g. Herein, the highest TPC value was found for *F. prolixa* aerial roots crude extract, with a value of 148 mg GAE/g. The TPCs of the last two extracts were measured presently as a first report.

Phenolic compounds are characterized by an aromatic ring with –OH or –OCH_3_ substituents. They have the ability to donate a hydrogen atom or electron because of their capacity to stabilize the formed phenol radical by resonance [6]. That is why a high number of phenolic compounds may suggest relatively radical scavenging and antioxidant capacity, investigated in the present work by conducting DPPH, FRAP, and AOP1 assays on the studied plant extracts.

### 3.3. DPPH Radical Scavenging Activity

**Figure 1:**
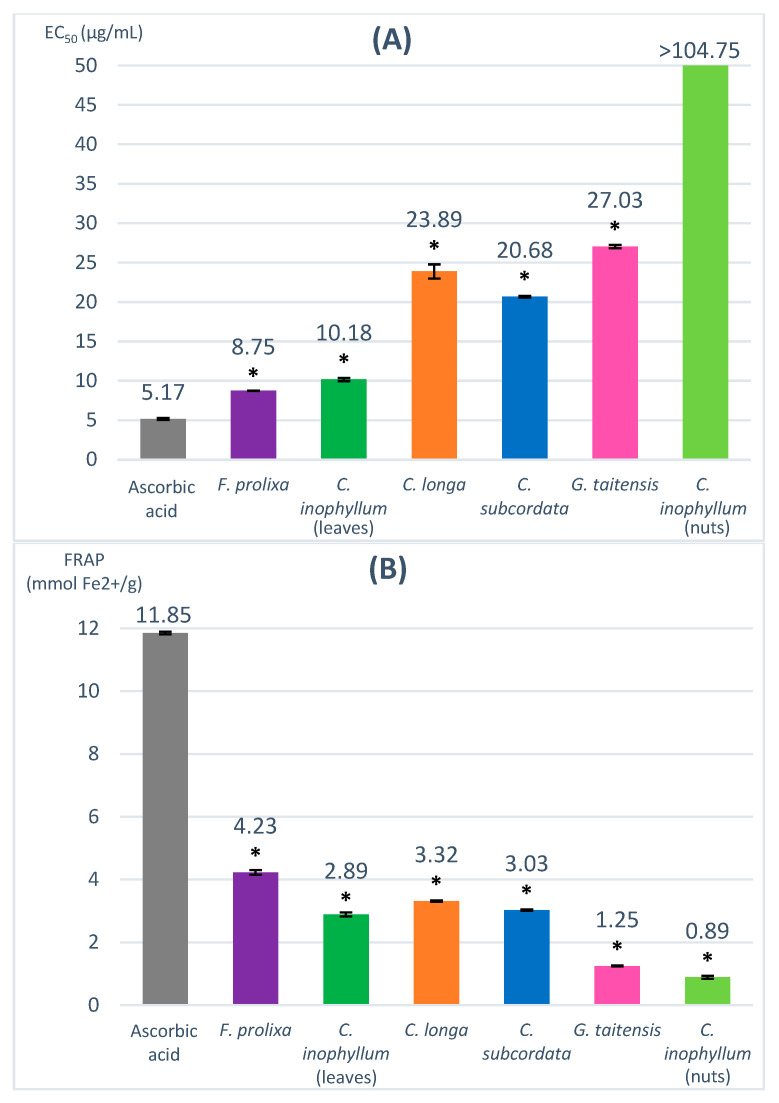
Radical scavenging and antioxidant capacity evaluated by DPPH (**A**) and FRAP assay (**B**). * = ANOVA followed by Dunnett’s multiple comparisons test results (*p* ≤ 0.01).

The DPPH (2,2-diphenyl-1-picrylhydrazyl) assay evaluates the capacity to scavenge the nitrogen radical, characterized by its deep violet color corresponding to UV/Vis spectrophotometry absorbance at 517 nm, into DPPH-H (yellow color). In the presence of hydrogen donor compounds, a correlated decrease in absorbance is observed. Results are expressed in EC_50_ (µg/mL), corresponding to the concentration of sample that decreases the DPPH absorbance by 50%, and compared to a positive control, ascorbic acid (EC_50_ = 5.17 µg/mL). A lower EC_50_ value means a more efficient scavenging activity. The obtained EC_50_ of studied plant extracts ranged from 8.75 to 27.03 µg/mL (Figure 1).

The used concentration range (5–100 µg/mL) did not allow us to determine the EC_50_ of *C. inophyllum* nuts extract, which may be correlated to a very low scavenging activity of this extract. Cassien et al. reported an EC_50_ of 432 µg/mL from a DPPH assay for cold-pressed *C. inophyllum* oil [21]. The leaves of *C. inophyllum* extract showed a higher scavenging activity with an EC_50_ of 10.18 µg/mL. The *C. longa* rhizomes extract had an EC_50_ of 23.89 µg/mL, in agreement with Sabir et al.’s results which reported similar results for an ethanolic extract of turmeric (27.2 μg/mL) [25]. Despite a low TPC value, *G. taitensis* flowers extract seemed to possess interesting radical scavenging activity (EC_50_ = 27.03 µg/mL), which may be due to non-phenolic constituents having radical scavenging capacity. Such properties had been previously reported in the *Gardenia* genus in *G. jasminoides* flowers [26,27]. *F. prolixa* crude extract had the lowest EC_50_ value (8.75 µg/mL), suggesting its highest scavenging capacity amongst the studied plant extracts.

### 3.4. Ferric-Reducing Antioxidant Power (FRAP) Assay

The FRAP (ferric-reducing antioxidant power) assay evaluates the capacity to reduce Fe^3+^ to Fe^2+^ in complex with TPTZ (2,4,6-tri(2-pyridyl)-s-triazine) inducing a blue color corresponding to UV/Vis spectrophotometry absorbance at 593 nm. The higher the FRAP values, the stronger the antioxidant activity. Ascorbic acid was used as a positive control (11.85 mmol Fe^2+^/g). The obtained FRAP value for our studied plant extracts ranged from 0.89–4.23 mmol Fe^2+^/g (Figure 1).

*C. inophyllum* nuts extract showed the lowest FRAP value (0.89 mmol Fe^2+^/g) and seemed to have very low antioxidant properties. The FRAP value of *C. inophyllum* leaves extract was higher (2.89 mmol Fe^2+^/g) than the obtained result for *C. inophyllum* nuts extract. Similar differences in antioxidant activity between leaves and nuts was reported by Hughes et al. for ethyl acetate and aqueous extract [18]. *C. subcordata* extract had a FRAP value of 3.03 mmol Fe^2+^/g. Only very few data are reported about the properties of this plant, but a previous study mentioned some antioxidant activity of ethanol leaves extracts in rat models [28]. *F. prolixa* extract showed the highest FRAP value (4.23 mmol Fe^2+^/g), indicating the best antioxidant capacity among the studied plant extracts.

In this study, the DPPH and FRAP assay results of the studied plant extracts are consistent with the above-obtained data of their TPC contents. Plant crude extracts with a high number of phenolic compounds, such as *F. prolixa*, seemed to have better antioxidant and scavenging properties. No study had been reported previously regarding these radical scavenging or antioxidant properties of *F. prolixa* extract. Another *Ficus* species, namely, *Ficus microcarpa*, was shown to have some antioxidant and scavenging properties, as reported by Ao et al., with an EC_50_ DPPH value of 6.8 µg/mL for a methanol extract of its aerial roots extract [29].

As among the presently studied five plant extracts, *F. prolixa* showed the most promising antioxidant and anti-radical scavenging activities, further investigations were performed on its crude extract.

### 3.5. Scavenging and Antioxidant Properties of Liquid/Liquid Extracts of F. prolixa

Liquid/liquid (L/L) extraction and fractionation were performed on crude extract of *F. prolixa* solubilized in water on a column Chromabond XTR using stepwise gradient solvents for elution of, respectively, hexane, dichloromethane, ethyl acetate, and butanol.

Hexane and dichloromethane fractions yields were very low due to the initial polarity of *F. prolixa* crude extract and so, scavenging and antioxidant properties were assessed only for ethyl acetate and butanol fractions. The obtained EC_50_ values from DPPH assay of these later fractions, respectively, 3.87 and 3.28 µg/mL for ethyl acetate and butanol extracts, were lower than those of ascorbic acid (Table 2). DPPH scavenging activity of fractions obtained from the methanolic aerial roots crude extract of *F. microcarpa*, respectively, for ethyl acetate extract (EC_50_ = 6.0 µg/mL) and butanol extracts (EC_50_ = 11.2 µg/mL), as reported by Ao et al. [27], suggested lower scavenging properties of *F. microcarpa* extracts compared to our results regarding *F. prolixa* fractions. The FRAP assay results of these *F. prolixa* fractions, respectively, of 9.36 and 9.18 mmol Fe^2+^/g for ethyl acetate and butanol fractions, were quite in the same range of the FRAP value of ascorbic acid (Table 2). Thus, these *F. prolixa* fractions should contain polar constituents with strong scavenging and antioxidant properties.

Aiming to evaluate and confirm the antioxidant effect of the *F. prolixa* extract on a cell model, AOP1 assay was then performed on keratinocytes.

### 3.6. Antioxidant Power Assay on F. prolixa Extract

Antioxidant Power 1 (AOP1) assay, performed on the HaCaT human keratinocyte model, allowed us to evaluate the antioxidant intracellular activity of tested samples. Using the patented Light-Up Cell System (LUCS) technology, cells were incubated in the culture medium with a photo-inducible fluorescent nucleic acid biosensor. The light application triggers the production of singlet oxygen which, in turn, causes the production of ROS, in a biochemical cascade linked to an increase of emitted fluorescence. Therefore, by measuring the fluorescence, this approach evaluated the ability of plant extracts to neutralize oxidative stress in cells [14].

**Figure 2:**
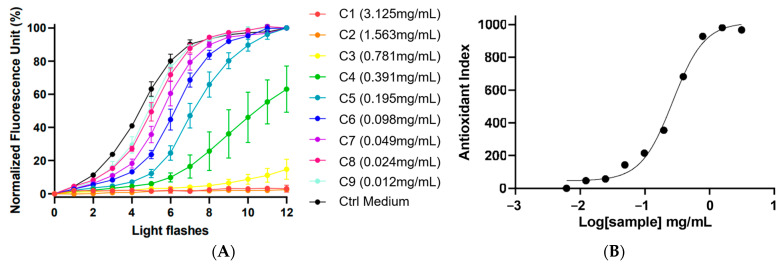
AOP1 assay of *F. prolixa* crude extract. (**A**) kinetic data: normalized fluorescence unit (%) obtained with increasing light flashes at each tested concentrations; (**B**) Dose–Response graph (EC_50_ = 268.0 µg/mL).

The kinetic graph (Figure 2) showed the % of fluorescence in relation to the number of light flashes at various extract concentrations of *F. prolixa* crude extract (from 0.012 mg/mL to 3.125 mg/mL). The black curve is the negative control (without added antioxidant), showing the cellular response to the induced production of ROS and the increasing % of fluorescence. Herein, *F. prolixa* crude extract showed a full direct antioxidant activity by neutralization of intracellular free radicals on human HaCaT cells. Any cytotoxic effects after 1 h were detected for any assayed concentrations below or equal to 25 mg/mL (max concentration tested). Dose–response curves showed a calculated antioxidant index according to logarithm (10) of the sample concentration (Figure 2). Efficacy concentration of *F. prolixa* crude extract had been measured and showed an EC_50_ value of 268.0 µg/mL on HaCaT cells. The EC_10_ of 77.6 µg/mL indicates the needed concentration to have an antioxidant activity. The EC_90_ of 924.6 µg/mL represents the concentration that neutralized 90% of the produced ROS. Taken as an example for comparison, resveratrol had an EC_50_ of 0.2621 µg/mL on this AOP1 assay on HaCaT (unpublished data). The obtained results for *F. prolixa* crude extract showed a good dose–response effect of the extract antioxidant activity on HaCaT cells.

This AOP1 assay demonstrated the capacity of *F. prolixa* extract to reduce oxidative stress and to provide a strong antioxidant activity on skin cells at non-cytotoxic concentrations. These data are promising results for potential skin topical applications of this plant extract.

### 3.7. Online RP HPLC DPPH Assay

In order to identify compounds responsible for the radical scavenging activity of the studied plant extracts, HPLC analysis was combined with DPPH online assay. Plant extract components were first separated and detected at 280 nm. This was then followed by the reaction of chromatographed components with DPPH solution, incorporated in the analytic system. Then, bleaching of the solution induced by active compounds was detected at 515 nm. Rutin was used as a positive control to determine the elution time shift between both detection systems.

**Figure 3:**
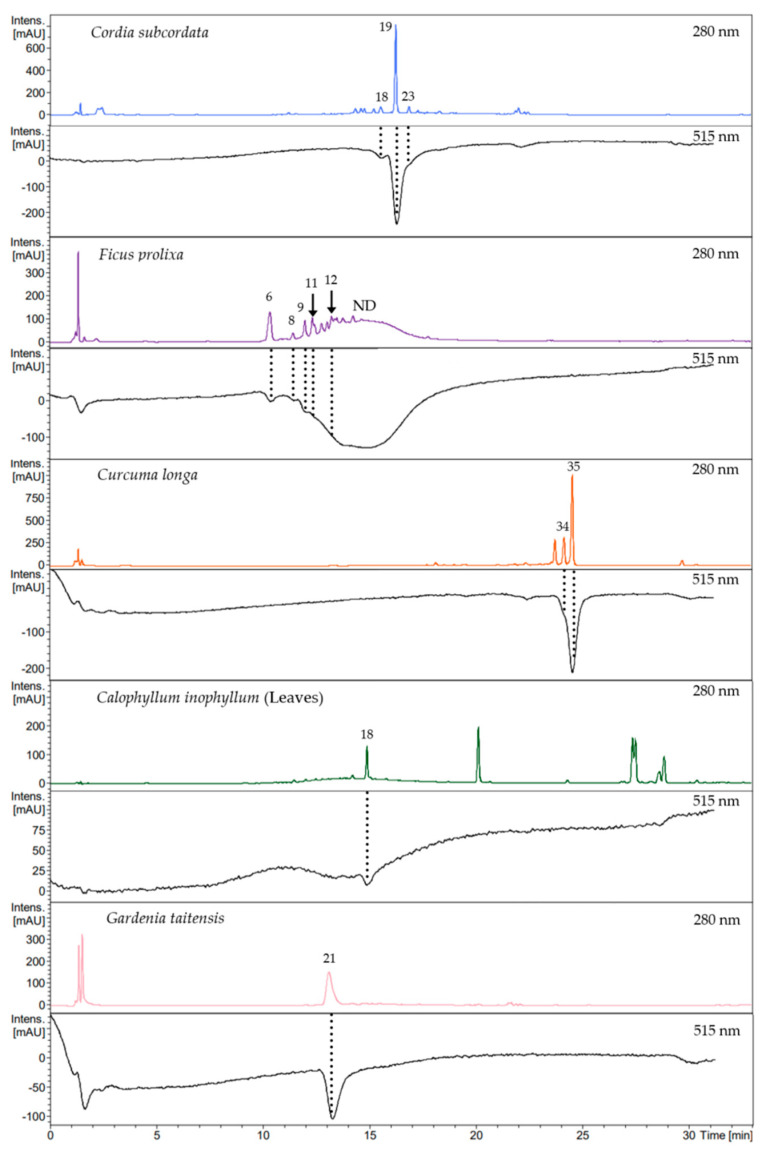
Chromatograms of Online RP HPLC DPPH assay. Crude extract profiles were recorded at 280 nm and their corresponding active compounds, shown in negative peaks, were recorded at 515 nm: chlorogenic acid (**6**), procyanidin type B (**8**), epicatechin (**9**), 5-*O*-caffeoylshikimic acid (**11**), procyanidin type C (**12**), quercetin-*O*-rhamnoside (**18**), rosmarinic acid (**19**), 3,5-di-*O*-caffeoyl-4-*O*-(3-hydroxy, 3-methyl)glutaroylquinic acid (**21**), lithospermate B (**23**), demethoxycurcumin enol form (**34**), curcumin enol form (**35**), ND: Not Determined.

This DPPH online analysis revealed that the scavenging activity of plant extracts, respectively, for *C. subcordata*, *C. longa*, *C. inophyllum* leaves, and *G. taitensis* was, for each, mostly due to one main compound (Figure 3), except for *F. prolixa* extract, which presented multiple compounds with scavenging properties. *C. inophyllum* nuts extract showed no decrease in UV/Vis spectrophotometry absorbance at 515 nm (Figure S1) which means that its constituents had no or very weak radical scavenging properties or the concentration of active compounds was too low to be detected. This finding was in agreement with Cassien et al. regarding scavenging activity of *C. inophyllum* oil extract. Some neoflavonoids content from *C. inophyllum* oil, especially inophyllum P, showed an EC_50_ of 26.2 µM from DPPH assay, but this compound represented only 0.05% of the extract [21].

By using a similar analytical method, those active compounds can be characterized by UHPLC-MS/MS analysis (Table 3).

### 3.8. UHPLC-MS/MS and Molecular Network

Mass tandem spectrometry was performed to study the phytochemical composition of the crude extracts. Spectral data were acquired in both positive and negative ionization modes using an LC-MS Q-TOF. Data were then processed via MZmine 3 and structured into molecular networks using GNPS. Finally, a total of 61 metabolites had been annotated (Table 3) by using reference standards, previous literature data, mass databases, spectral prediction, and molecular networking. Herein, the identification levels of confidence of Schymanski et al. were applied to classify annotated compounds [30]: starting with level 3 (L3) for tentative candidate, followed by level 2 for probable structure identified by diagnostic evidence (L2b) or by library spectrum match (L2a), and then level 1 (L1) for confirmed structure by the use of reference standard compound or by NMR.

**Figure 4:**
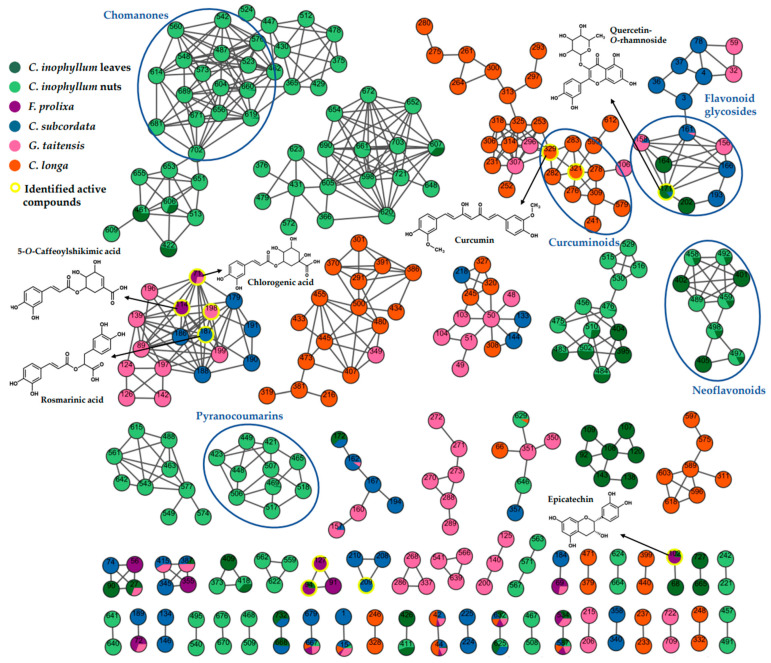
Molecular Network cluster created with GNPS using spectral data of crude extracts in positive mode (self-loop nodes removed). Node colors represent repartition in plant extracts: *C. inophyllum* leaves (dark green), *C. inophyllum* nuts (light green), *F. prolixa* (purple), *C. subcordata* (blue), *G. taitensis* (pink), *C. longa* (orange). Node numbers correspond to MZmine numbers in positive mode as shown in Table 3 and in LC/MS data at: https://doi.org/10.5281/zenodo.8300733 (accessed on 1 September 2023).

The resulting molecular networks (Figure 4 and Figure S2) revealed big green clusters, suggesting that some chemical classes were specific to *C. inophyllum*. A difference in composition and amount between leaves and nuts was noticed. Metabolites from *C. inophyllum* extracts were mainly identified by standards from our internal database. Two light green clusters showed that some pyranocoumarins and chromanones are specific to nuts. Indeed, pyranocoumarins such as tamanolide (**53**), tamanolide E (**44**) and C (**51**), and calanolide D (**41**) had been identified with a high level of confidence (L2a) thanks to standards from our internal database. Otherwise, calanolide A (**42**) and B (**45**) were annotated as tentative for identification (L3). These two isomer compounds had a structure close to calanolide D that only differs from a hydroxyl group due to ketone function. According to these data, other identifications were suggested, 12-oxocalanolide A or B (**40**), as isomers of calanolide D, and 12-methoxycalanolide A (**52**) and B (**54**) [31]. Among chomanones, inocalophyllin B methyl ester (**61**) was identified, showing the same fragmentation behavior as inocalophyllin B (**55**) but with a difference of 14 uma due to a methyl group. Likewise, the structural difference between inocalophyllin A (**56**) and inocalophyllin B was consistent with MS spectral information and expressed by a difference of 34 uma [32]. Caledonic acid (**47**) is described as a tentative of identification as it is the only known compound in Calophyllaceae with a molecular formula of C_27_H_38_O_6_. Neoflavonoids (4-phenylcoumarins or Ar-C3-Ar) like calophyllolide (**49**) inophyllum E (**48**) and its isomer soulattrolone (**43**) were found in both plant part extracts. Some metabolites were identified in leaves only: jacareubin (**36**), inophyllum G (**37**), and tomentolide A (**38**) [33,34,35]. Flavonoids and derivatives were also identified as major constituents in leaves: quercitrin-*O*-rhamnoside (**18**), procyanidin type B (**8**) [36], amentoflavone (**30**) [19], and epicatechin (**9**). These compounds are known for their antioxidant properties and could be responsible for the activity of this extract. As they were only found in leaves extracts, this difference of composition between part of plants could explain the relative absence or very low levels of antioxidant activity in nuts extract.

This molecular network also showed an orange cluster composed of curcuminoids. These polyphenol compounds, specific to *Curcuma* species, are major compounds in *C. longa* rhizomes, with an amount of up to 2590 mg/100 g for curcumin [37]. Curcumin, demethoxycurcumin, and bisdemethoxycurcumin were identified in both keto and enol forms in *C. longa* extract. As described by Jia et al., the presence of the β-diketone system in curcuminoid creates a keto–enol tautomerism. The keto form can be distinguished by its lower peak area, earlier retention time, and difference of fragmentation in negative ion mode [38].

In *G. taitensis* flowers extract, various compounds were identified: some iridoids like gardenoside (**5**) and geniposide (**10**), flavonoids such as rutin (**14**) and quercetin-*O*-hexose (**15**), and some phenolic acids as chlorogenic acid (**6**) and 3,5-di-*O*-caffeoyl-4-*O*-(3-hydroxy, 3-methyl)glutaroylquinic acid (**21**). Spectral MS/MS data of these compounds were consistent with those mentioned by Guo et al. in *G. jasminoides* flowers [39]. A terpene compound was identified as 7,8,11-trihydroxyguai-4-en-3-one-8-*O*-β-D-glucopyranoside (**16**), also reported in *G. jasminoides* [40].

As few data are available regarding *F. prolixa* and *C. subcordata* extract constituents, this molecular network facilitated the identification process. Identification of compounds from well-known plants with phytochemistry LC-MS data helped to confirm the chemical class of compounds in the cluster from plants of unknown composition. Moreover, some metabolites found in various plants, and that have been identified in one of them in previous studies, helped to confirm their identification and occurrence in the other less known plants.

Compounds identified in *C. subcordata* extract in the present work were described for the first time for this species. Listhospermoside (**1**) is a cyanoglucoside mentioned by Sosa et al. in other Boraginaceae: *Lithospermum purpureo-caeruleum* and *Lithospermum oficinale* [41]. Flavonoid glycosides such as quercetin derivatives were also detected. They had been previously isolated in *Cordia* species and known to possess radical scavenging properties [42,43]. Rosmarinic acid (**19**) and lithospermate B (**23**), a rosmarinic acid dimer, were identified. These polyphenols had also been isolated in various Boraginaceae such as *Lithospermum erythrorhizon* [44] and *Cordia sebestana* [45].

Metabolites identified in *F. prolixa* were mentioned for the first time in this species: chlorogenic acid (**6**) and cryptochlorogenic acid (**7**), procyanidin B1 or B2 (**8**) and type C (**12**), epicatechin (**9**), and 5-*O*-caffeoylshikimic acid (**11**). In the same way, Ao et al. had isolated epicatechin, procyanidin B1, and chlorogenic acid in another *Ficus* species, specifically *F. microcarpa*, and reported their radical scavenging activity by DPPH assay [46].

**Table 3:** Metabolites identified in crude extracts (ethanol 70%) of five Polynesian plants, *C. inophyllum* leaves, *C. inophyllum* nuts, *F. prolixa*, *C. subcordata*, *G. taitensis*, and *C. longa* analyzed by UHPLC-MS/MS (Qtof) in both negative and positive ionization modes. Metabolites are sorted by retention times (RT).

N°	Annotation	Molecular Formula	RT (min)	IC	MS				MSMS		Ref	Plants
					MM N° +	[M+H]^+^ (Error in ppm)	MM N° −	[M-H]^−^ (Error in ppm)	[M+H]^+^ (Relative Intensity in %)	[M-H]^−^ (Relative Intensity in %)		
**1**	Lithospermoside	C_14_H_19_NO_8_	0.62	L2b	3	330.1184 (+0.2)	4	328.1037 (−0.3)	168.0653 (100); 122.0599 (33); 105.0333 (20); 330.1186 (15)	148.0406 (100); 130.0301 (95); 283.2646 (88); 146.0243 (81); 161.0450 (55)	[41,47,48]	*C. subcordata*
**2**	Pantothenic acid	C_9_H_17_NO_5_	1.04	L2b	21	220.1183 (+1.6)	ND	ND	90.0552 (100); 202.1067 (37); 116.0349 (35); 184.0964 (32); 103.0750 (24); 95.0494 (22)	ND	[49]	*F. prolixa* *C. subcordata*
**3**	Prunasin amide	C_14_H_19_NO_7_	1.39	L3	36	314.124 (+1.8)	ND	ND	152.0708 (100); 107.0501 (15); 194.0799 (10); 296.1128 (10); 134.0601 (9); 314.1247 (7)	ND	[50]	*C. subcordata*
**4**	Sinapic acid	C_11_H_12_O_5_	1.80	L2a	51	225.0761 (+1.6)	ND	ND	91.0542 (100); 147.0439 (93); 119.0490 (80); 95.0492 (34); 175.0387 (32); 123.0441 (19); 189.0545 (16)	ND	[51]	*G. taitensis*
**5**	Gardenoside	C_17_H_24_O_11_	1.80	L2a	ND	ND	29	403.1245 (−0.2)	ND	127.0403 (100); 241.0699 (70); 177.0557 (41); 89.0246 (34)	[39,52,53]	*G. taitensis*
**6**	Chlorogenic acid (5-CQA)	C_16_H_18_O_9_	3.46	L1	71	355.1026 (+0.7)	46	353.0877 (−0.3)	163.0388 (100); 135.0437 (12); 145.0283 (7); 117.0334 (4)	191.0559 (100); 85.0294 (4); 127.0403 (2)	[39,46]	*F. prolixa* *G. taitensis*
**7**	Cryptochlorogenic acid (4-CQA)	C_16_H_18_O_9_	4.22	L2a	89	355.1032 (+2.4)	58	353.0872 (−1.7)	163.0394 (00); 135.0443 (14); 145.0286 (8); 193.0501 (5)	173.0452 (100); 135.0446 (88); 179.0359 (81); 191.0552 (65)	[54]	*F. prolixa* *G. taitensis*
**8**	Procyanidin B1 or B2	C_30_H_26_O_12_	5.06	L2a	94	579.1505 (+1.4)	64	577.1351 (−0.1)	127.0390 (100); 139.0390 (43); 287.0551 (35); 163.0390 (34); 289.0712 (33); 291.0855 (33); 271.0605 (30); 275.0543 (24); 247.0592 (23)	289.0714 (100); 407.0765 (86); 125.0245 (47); 425.0857 (46); 577.1339 (31); 426.0897 (24); 451.1021 (24); 245.0822 (20)	[36,46,55,56]	*F. prolixa* *C. inophyllum* leaves
**9**	Epicatechin	C_15_H_14_O_6_	5.41	L2a	102	291.0867 (+1.3)	70	289.0716 (−0.6)	139.0390 (100); 123.0440 (62); 147.0440 (16); 207.0650 (14); 165.0543 (12)	123.0450 (100); 109.0285 (81); 137.0235 (52); 151.0390 (52); 245.0807 (41); 121.0292 (40); 125.0237 (39); 149.0247 (38); 205.0508 (36)	[46,57,58]	*F. prolixa* *C. inophyllum* leaves
**10**	Geniposide	C_17_H_24_O_10_	5.43	L2a	ND	[M+NH_4_]^+^ 406.1709	ND	ND	209.0810 (100); 149.0596 (75); 227.0913 (46); 121.0649 (39); 177.0547 (38)	ND	[39,59]	*G. taitensis*
**11**	5-*O*-caffeoylshikimic acid	C_16_H_16_O_8_	5.65	L2a	114	337.0917 (−0.3)	80	335.0771 (−0.4)	163.0387 (100); 135.0440 (16); 145.0281 (7); 117.0336 (5); 89.0384 (3)	135.0450 (100); 179.0349 (81); 161.0245 (27); 133.0293 (16); 93.0342 (9)	[60]	*F. prolixa*
**12**	Procyanidin type C	C_45_H_38_O_18_	6.26	L2a	127	867.2144 (1.5)	96	865.1986 (+0.1)	289.0705 (100); 247.0599 (58); 127.0388 (49); 275.0544 (35); 163.0385 (34); 409.0918 (32); 579.1512 (31)	865.1976 (100); 287.0559 (78); 407.0757 (67); 289.0716 (65); 577.1346 (60); 575.1208 (48); 425.0879 (48); 125.0242 (43); 451.1039 (32); 413.0849 (30)	[61,62]	*F. prolixa*
**13**	Icariside B5	C_19_H_32_O_8_	6.70	L2b	143	389.2179 (−0.5)	ND	ND	209.1531 (100); 191.1436 (55); 149.0962 (35); 173.1327 (27); 163.1470 (23)	ND	[63]	*C. inophyllum* leaves
**14**	Rutin	C_27_H_30_O_16_	7.45	L2a	158	611.1609 (+0.4)	122	609.1465 (+0.6)	303.0504 (100); 129.0551 (8); 85.0285 (7); 465.1044 (4)	300.0275 (100); 609.1464 (85); 271.0254 (3); 178.9994 (2); 151.0036 (1); 255.0309 (1)	[39,64,65,66]	*C. subcordata* *G. taitensis*
**15**	Quercetin-*O*-hexose	C_21_H_20_O_12_	7.58	L2a	161	465.1031 (+0.7)	127	463.088 (−0.4)	303.0500 (100); 85.0282 (7); 145.0494 (5); 127.0389 (4); 97.0288 (3); 91.0396 (1)	300.0272 (100); 463.0879 (54); 271.0243 (25); 255.0299 (10)	[39,67,68]	*C. subcordata* *G. taitensis*
**16**	7,8,11-trihydroxyguai-4-en-3-one-8-*O*-β-D-glucopyranoside	C_21_H_34_O_9_	7.86	L2a	165	431.2282 (+1.5)	133	429.2129 (−0.2)	269.1851 (100); 251.1644 (57); 233.1533 (44); 163.1117 (23); 137.0962 (15);	174.9579 (100); 209.1232 (92)	[40,69]	*G. taitensis*
**17**	Quercetin 3-malonylglucoside	C_24_H_22_O_15_	8.02	L2a	166	551.104 (+1.5)	137	549.0888 (+0.4)	303.0502 (100); 127.0387 (14); 159.0293 (7); 145.0496 (7); 109.0284 (6)	300.0283 (100); 505.0992 (67); 271.0268 (1)	[70]	*C. subcordata*
**18**	Quercetin- *O*-rhamnoside	C_21_H_20_O_11_	8.34	L2a	171	449.1081 (+0.6)	145	447.093 (−0.6)	303.0500 (100); 85.0281 (21); 129.0543 (15); 71.0488 (8)	300.0271 (100); 447.0929 (51); 271.0242 (26); 255.0295 (13)	[36,71,72]	*C. subcordata* *C. inophyllum* leaves
**19**	Rosmarinic acid	C_18_H_16_O_8_	8.77	L2a	187	361.0922 (+1.1)	153	359.0772 (−0.1)	163.0391 (100); 135.0441 (19); 139.0390 (9); 145.0287 (7); 181.0495 (5); 117.0337 (4); 89.0385 (2)	161.0242 (100); 197.0454 (36); 135.0450 (30); 133.0294 (28); 179.0349 (20); 123.0448 (14); 72.9931 (13)	[45,73,74]	*C. subcordata*
**20**	Kaempferol *O*-malonylglucoside	C_24_H_22_O_14_	8.86	L2a	193	535.1092 (+1.8)	ND	ND	287.0553 (100); 127.0391 (14); 145.0495 (7); 159.0287 (6); 109.0287 (6)	ND	[75]	*C. subcordata*
**21**	3,5-di-*O*-caffeoyl-4-*O*-(3-hydroxy, 3-methyl)glutaroylquinic acid	C_31_H_32_O_16_	9.17	L2a	198	661.1764 (+0.1)	163	659.1616 (−0.2)	163.0393 (100); 301.0927 (5); 135.0445 (2); 355.1032 (2); 337.0919 (2); 145.0286 (2)	497.1298 (100); 335.0771 (40); 191.0559 (37); 161.0454 (35); 335.0974 (21); 659.1616 (19); 353.0875 (16)	[39,76,77]	*G. taitensis*
**22**	Kaempferol- *O*-rhamnoside	C_21_H_20_O_10_	9.21	L2a	202	433.1127 (−0.5)	166	431.0977 (−1.6)	287.0549 (100); 85.0279 (25); 129.0540 (22)	285.04 (100); 255.0292 (40); 227.0367 (38); 431.0974 (37)	[78]	*C. inophyllum* leaves
**23**	Lithospermate B	C_36_H_30_O_16_	9.64	L2a	209	719.1613 (+0.9)	167	717.1456 (−0.7)	181.0496 (100); 323.0553 (71); 295.0606 (53); 139.0390 (36); 521.1081 (34)	321.0399 (100); 519.0931 (97); 339.0509 (48); 295.0600 (17)	[79,80]	*C. subcordata*
**24**	Curcumalongin A	C_20_H_16_O_6_	11.63	L2a	233	353.1024 (+1.2)	191	351.0877 (+0.8)	353.1022 (100); 147.0446 (22); 153.0546 (18); 166.0260 (16); 149.0233 (9); 121.0287 (8); 150.0313 (7); 338.0804 (6)	351.0880 (100); 279.0660 (94); 308.0698 (90); 336.0657 (73); 291.0671 (63); 143.0505 (44)	[38]	*C. longa*
**25**	Bisdemethoxycurcumin (keto form)	C_19_H_16_O_4_	11.66	L2a	ND	309.1126 (+1.5)	192	307.0979 (+1.0)	147.0442 (100); 119.0490 (22); 91.0543 (6)	145.0294 (100); 119.0505 (65); 117.0346 (49); 161.0611 (26); 143.0502 (16); 214.9273 (10)	[38]	*C. longa*
**26**	Curcumalongin B	C_21_H_18_O_7_	11.98	L2a	237	383.1129 (+1.0)	200	381.0985 (+1.4)	383.1129 (100); 153.0546 (13); 149.0233 (7); 177.0550 (6); 163.0385 (6); 294.0881 (5); 145.0287 (5)	381.0985 (100); 366.0756 (63); 277.0505 (36); 309.0773 (34); 295.0609 (31); 267.0681 (24); 338.005 (23)	[38]	*C. longa*
**27**	Demethoxycurcumin (keto form)	C_20_H_18_O_5_	12.06	L2a	ND	339.1230 (+0.9)	202	337.1083 (+0.5)	177.0547 (100); 147.0441 (66); 145.0285 (32); 119.0495 (11)	145.0293 (100); 175.0404 (77); 160.0161 (57); 119.0501 (55); 117.0353 (45)	[38]	*C. longa*
**28**	Curcumin (keto form)	C_21_H_20_O_6_	12.44	L2a	ND	369.1337 (+1.2)	207	367.1195 (+2.1)	177.0550 (100); 145.0287 (39); 117.0336 (12)	175.0404 (100); 160.0172 (83); 134.0378 (28); 132.0218 (23);	[38]	*C. longa*
**29**	Centaureidin	C_18_H_16_O_8_	12.86	L2b	250	361.0924 (+1.7)	211	359.0773 (+0.2)	361.0923 (100); 303.0501 (53); 331.0439 (17); 346.0687 (13); 345.0618 (11); 328.0593 (9)	344.0550 (100); 329.0307 (80); 286.0119 (80); 301.0378 48); 359.0772 (39); 258.0170 (37)	[81]	*G. taitensis*
**30**	Amentoflavone	C_30_H_18_O_10_	13.54	L2a	257	539.0986 (+2.5)	216	537.0834 (+1.3)	539.0986 (100); 403.0453 (8); 377.0662 (7); 387.0876 (3); 497.0882 (2); 421.0565 (2); 335.0548 (2)	537.0833 (100); 375.0514 (80); 417.0616 (22); 376.0545 (19); 331.0612 (12)	[19,82]	*C. inophyllum* leaves
**31**	2,3-dihydro amentoflavone	C_30_H_20_O_10_	14.01	L2a	274	541.1136 (+1.3)	231	539.0983 (+0.2)	389.1039 (100); 541.1131 (63); 153.0182 (41); 171.0293 (28)	413.0663 (100); 387.0870 (76); 539.0982 (46); 537.0840 (29); 251.0355 (26); 225.0551 (25)	[83,84]	*C. inophyllum* leaves
**32**	Chikusetsusaponin iva	C_42_H_66_O_14_	14.54	L2a	ND	ND	239	793.4371 (−1.1)	ND	793.4372 (100); 631.3829 (6); 569.3832 (2)	[85]	*G. taitensis*
**33**	Bisdemethoxycurcumin (enol form)	C_19_H_16_O_4_	15.69	L2a	309	309.1129 (+2.5)	280	307.0978 (+0.7)	147.0445 (100); 225.0918 (46); 119.0497 (39); 91.0546 (12)	119.0505 (100); 143.0504 (25); 187.0401 (7)	[38,86]	*C. longa*
**34**	Demethoxycurcumin (enol form)	C_20_H_18_O_5_	16.11	L2a	321	339.1238 (+3.2)	292	337.1087 (+1.6)	147.0446 (100); 177.0553 (85); 255.1026 (68); 145.0291 (41); 119.0497 (29); 117.0341 (18); 223.0763 (16)	119.0505 (100); 134.0375 (12); 158.0374 (11); 173.0611 (10); 143.0503 (9); 217.0509 (6); 149.0609 (6); 202.0272 (4)	[38,86]	*C. longa*
**35**	Curcumin (enol form)	C_21_H_20_O_6_	16.53	L2a	329	369.134 (+2.0)	300	367.1187 (0)	177.0549 (100); 145.0287 (54); 285.1127 (30); 117.0338 (18); 161.0603 (12)	134.0374 (100); 149.0609 (55); 173.0609 (24); 158.0375 (22); 217.0509 (12)	[38,86]	*C. longa*
**36**	Jacareubin	C_18_H_14_O_6_	16.80	L2a	335	327.0869 (+1.8)	305	325.0721 (+1.0)	327.0871 (100); 273.0407 (33); 257.0460 (13); 285.0403 (11)	325.0720 (100); 309.0405 (23); 295.0257 (9); 310.0466 (8); 267.0306 (4)	[87,88]	*C. inophyllum* leaves
**37**	Inophyllum G	C_25_H_24_O_5_	20.27	L3	396	405.1701 (+1.6)	350	403.155 (−0.2)	387.1601 (100); 349.1072 (33); 405.1701 (27) 311.0548 (19); 345.1122 (18)	403.1554 (100); 347.0925 (46); 348.0968 (11); 303.1034 (9)	[34]	*C. inophyllum* leaves
**38**	Tomentolide A	C_25_H_22_O_5_	20.43	L3	401	403.1547 (+1.7)	ND	ND	403.1547 (100); 347.0914 (77); 365.1015 (12); 293.0432 (11); 171.0448 (10)	ND	[35]	*C. inophyllum* leaves
**39**	Calophyllic acid	C_25_H_24_O_6_	20.55	L2a	409	421.1653 (+1.7)	353	419.1496 (−1.0)	403.1538 (100); 347.0913 (56); 377.1746 (46); 321.1121 (31)	375.1592 (100); 319.0958 (12); 419.1489 (11)	[34] ID	*C. inophyllum* nuts *C. inophyllum* leaves
**40**	12-oxocalanolide A or B	C_22_H_24_O_5_	20.64	L3	423	369.1695 (−0.7)	ND	ND	369.1695 (100); 285.1121 (45); 341.1746 (19); 313.1056 (14); 257.1165 (9); 243.0637 (8)	ND	[21]	*C. inophyllum* nuts
**41**	Calanolide D	C_22_H_24_O_5_	21.06	L2a	448	369.1694 (−0.7)	ND	ND	369.1695 (100); 285.1121 (29); 341.1739 (21); 313.1075 (9); 189.1273 (9); 257.1155 (7)	ND	[21] ID	*C. inophyllum* nuts
**42**	Calanolide A	C_22_H_26_O_5_	21.15	L3	456	371.1847 (−1.6)	ND	ND	353.1741 (100); 371.1847 (17); 311.1270 (12); 283.0966 (7); 325.1800 (6)	ND	[21,31]	*C. inophyllum* nuts
**43**	Soulattrolone	C_25_H_22_O_5_	21.16	L2b	458	403.154 (0)	ND	ND	403.1534 (100); 347.0911 (64); 365.1017 (8); 293.0439 (5); 319.0950 (5)	ND	[89]	*C. inophyllum* nuts *C. inophyllum* leaves
**44**	Tamanolide E	C_23_H_26_O_5_	21.28	L2a	469	383.1855 (+0.5)	ND	ND	383.149 (100); 327.1226 (51); 299.1277 (27); 355.1902 (25); 328.1254 (11); 269.0804 (9); 281.1163 (7)	ND	ID	*C. inophyllum* nuts *C. inophyllum* leaves
**45**	Calanolide B	C_22_H_26_O_5_	21.33	L3	476	371.1855 (+0.5)	ND	ND	353.1745 (100); 371.1849 (42); 311.1278 (16); 325.1794 (8); 283.1321 (7)	ND	[21,31]	*C. inophyllum* nuts
**46**	Inophyllum A or D	C_25_H_24_O_5_	21.47	L2b	484	405.1691 (−1.4)	ND	ND	387.1595 (100); 405.1701 (17); 345.1129 (15); 317.0821 (21)	ND	[34]	*C. inophyllum* nuts *C. inophyllum* leaves
**47**	Caledonic acid	C_27_H_38_O_6_	21.50	L3	487	459.2737 (−0.9)	389	457.2578 (−3.9)	275.1275 (100); 335.1485 (75); 317.1380 (69); 233.0804 (24); 336.1520 (15)	457.2573 (100); 315.1587 (80); 301.1425 (52); 413.2671 (22)	[90]	*C. inophyllum* nuts
**48**	Inophyllum E	C_25_H_22_O_5_	21.54	L2a	492	403.154 (0)	ND	ND	403.1537 (100); 347.0911 (61); 387.1580 (7); 293.0443 (7); 365.1018 (6); 319.0961 (5)	ND	[21,34,91] ID	*C. inophyllum* nuts *C. inophyllum* leaves
**49**	Calophyllolide	C_26_H_24_O_5_	21.64	L2a	497	417.1696 (−0.1)	ND	ND	417.1692 (100); 361.1066 (57); 331.0599 (14); 362.1099 (13); 329.0803 (11)	ND	[34,91] ID	*C. inophyllum* nuts *C. inophyllum* leaves
**50**	Inophyllum P	C_25_H_24_O_5_	21.70	L2a	510	405.1688 (−2.1)	ND	ND	405.1676 (100); 387.1594 (33); 345.1129 (6); 317.0815 (4)	ND	[21,34,91] ID	*C. inophyllum* nuts *C. inophyllum* leaves
**51**	Tamanolide C	C_23_H_26_O_5_	21.69	L2a	506	383.1851 (−0.5)	ND	ND	383.1851 (100); 355.1904 (56); 299.1277 (43); 281.1175 (15); 287.1278 (14)	ND	ID	*C. inophyllum* nuts
**52**	12-Methoxycalanolide A	C_23_H_28_O_5_	21.81	L3	516	385.2007 (−0.7)	ND	ND	367.1905 (100); 385.2007 (23); 339.1952 (19); 295.1326 (14)	ND	[31]	*C. inophyllum* nuts
**53**	Tamanolide	C_24_H_28_O_5_	21.85	L2a	517	397.2007 (−0.6)	ND	ND	397.2008 (100); 369.2056 (35); 313.1432 (26); 341.1381 (23); 370.2091 (10); 339.1590 (8); 283.0964 (6); 245.0806 (6)	ND	[21] ID	*C. inophyllum* nuts
**54**	12-Methoxycalanolide B	C_23_H_28_O_5_	21.97	L3	530	385.2006 (−0.9)	ND	ND	367.1901 (100); 385.2006 (59); 339.1950 (25); 295.1327 (13)	ND	[31]	*C. inophyllum* nuts
**55**	Inocalophyllin B 1	C_32_H_46_O_6_	23.61	L2a	604	527.3363 (−0.8)	461	525.3205 (−3.2)	335.1487 (100); 275.1276 (84); 317.1382 (73); 318.1415 (16); 276.1310 (15); 233.0805 (10)	525.3205 (100); 383.2219 (36); 369.2061 (20); 481.3322 (18)	[32] ID	*C. inophyllum* nuts
**56**	Inocalophyllin A	C_35_H_44_O_6_	23.64	L2b	606	561.3209 (−0.3)	466	559.3056 (−1.6)	369.1333 (100); 351.1227 (64); 309.1122 (56); 221.0808 (53); 233.0809 (27)	559.3049 (100); 355.1907 (19); 323.1283 (16); 446.2451 (13); 471.3243 (10);	[32]	*C. inophyllum* nuts *C. inophyllum* leaves
**57**	Inocalophyllin B 2	C_32_H_46_O_6_	23.88	L2a	619	527.3373 (+1.1)	473	525.3212 (−1.8)	317.1385 (100); 335.1496 (76); 275.1281 (75); 336.1526 (30); 276.1314 (25); 69.0697 (24); 233.0810 (20)	525.3206 (100); 383.2216 (56); 369.2057 (33)	[32] ID	*C. inophyllum* nuts *C. inophyllum* leaves
**58**	Linoleic acid	C_18_H_32_O_2_	24.03	L2a	629	281.2477 (+0.7)	484	279.2322 (−2.7)	97.1011 (100); 83.0851 (69); 95.0857 (64); 109.1017 (55)	279.2325 (100); 146.9580 (1)	[92]	*C. inophyllum* nuts
**59**	Inocalophyllin B 3	C_32_H_46_O_6_	24.60	L2a	660	527.3368 (+0.2)	498	525.3218 (−0.7)	335.1492 (100); 275.1279 (92); 317.1384 (89); 276.1313 (20); 318.1418 (18); 459.2743 (17); 233.0807 (13); 69.0698 (13)	525.3209 (100); 333.1338 (50); 387.1805 (13); 334.1376 (12); 219.0658 (12)	[32] ID	*C. inophyllum* nuts
**60**	Pheophorbide A	C_35_H_36_N_4_O_5_	24.73	L2b	668	593.2764 (+0.9)	ND	ND	593.2761 (100); 533.2560 (18); 534.2571 (26); 460.2277 (3);	ND	[93]	*C. inophyllum* leaves *C. subcordata*
**61**	Inocalophillin B methyl ester	C_33_H_48_O_6_	25.15	L3	689	541.3519 (−0.9)	520	539.3368 (−1.9)	349.1642 (100); 331.1539 (87); 289.1434 (86)	539.3375 (100); 347.1490 (51); 348.1534 (14); 303.1585 (14)	[32]	*C. inophyllum* nuts

IC: Identification confidence. L1: reference standard or NMR. L2a: library spectrum match. L2b: diagnostic evidence. L3: tentative candidate. MM N°: MZmine Number. ND: Not detected. ID: Internal Database.

Using the same analytical method on both Online RP HPLC DPPH assay and LC-MS/MS analysis, the obtained chromatograms were consistent and the previously annotated compounds led to the identification of active metabolites. Thus the scavenging activity of crude extracts was mainly due to: quercetin-*O*-rhamnoside (**18**) in *C. inophyllum* leaves; rosmarinic acid (**19**) in *C. subcordata* leaves; chlorogenic acid (**6**), procyanidins type B (**8**) and C (**12**), epicatechin (**9**), and 5-*O*-caffeoylshikimic acid (**11**) in aerial roots of *F. prolixa*; curcumin (**35**) in *C. longa* rhizomes; 3,5-di-*O*-caffeoyl-4-*O*-(3-hydroxy, 3-methyl)glutaroylquinic acid (**21**) in *G. taitensis* flowers (Figure 3). According to the structure–activity relationships reported in the literature by Truzzi et al., hydroxycinnamic acids, especially caffeic acid derivatives, and flavonoids such as epicatechin and quercetin, are among the strongest scavengers [94]. This fact may explain the high scavenging capacity of *F. prolixa*, *C. inophyllum* leaves, and *C. subcordata* extracts within such compounds. Their strong scavenging activity could also be related to the amount of these compounds in these latter plant extracts. Identification of these bioactive phenolic molecules highlighted the antioxidant properties of these plant extracts.

The identified active compounds in *F. prolixa* extract could be correlated with its intracellular antioxidant activity on HaCaT cells. Indeed, various studies evaluated activities of these well-known antioxidant molecules on skin cells. Protective effects on HaCaT human keratinocytes against UV-induced oxidative damage were demonstrated by procyanidin fractions from *Vitis vinifera* [95]. In the same way, epicatechin increased the viability of UVB-irradiated HaCaT cells [96]. Chlorogenic acid and other caffeoyl derivatives extracted from *Ficus dubia* showed radical scavenging activity on keratinocytes [97]. Moreover, chlorogenic acid also reduced ROS production and HaCaT cell deaths when exposed to airborne particulate matters [98]. The pool of bioactive compounds with antioxidant potential in *F. prolixa* aerial roots make this extract a promising ingredient for skin care.

## 4. Conclusions

UHPLC-MS/MS and a molecular network approach enabled the characterization of the chemical composition of five Polynesian plants used in traditional medicine and skin care. This network led to the identification of 61 metabolites. Compounds annotated for *F. prolixa* and *C. subcordata* were described for the first time in these two indigenous Polynesian trees. As far as we are aware, no previous study had been reported regarding their phytochemical content.

Despite some limits of the analytical method, interesting phytochemical results were obtained on the studied plant extracts. Actually, common MS databases used for annotation are not exhaustive and do not allow for the identification of all detected compounds with high confidence levels. Moreover, characterization of isomer compounds, and especially stereoisomers, is limited by the use of LC-MS/MS data analysis, as their mass spectra fragmentation patterns can hardly be differentiated.

Further investigation could be performed to identify more active compounds in *F. prolixa* by the use of different HPLC analytical methods aiming at a better separation of analytes or allowing for checks of different classes of metabolites which were not found in the present work.

The performed DPPH and FRAP assays on the five studied plant extracts revealed the radical scavenging activity and the antioxidant activity of, respectively, *C. inophyllum* leaves, *F. prolixa* aerial roots, *C. subcordata* leaves, *G. taitensis* flowers, and *C. longa* rhizomes. DPPH online assay allowed the identification of phenolic active compounds such as quercetin-*O*-rhamnoside, rosmarinic acid, chlorogenic acid, procyanidin type B and C, epicatechin, 5-*O*-caffeoylshikimic acid, and curcumin as responsible for the antiradical scavenging properties of the plant extracts. Further investigations were performed on *F. prolixa* extract, considered as the most active one from the five studied plant extracts. AOP1 assay confirmed its intracellular antioxidant activity on a HaCaT human keratinocyte model. Moreover, DPPH and FRAP assays performed on L/L extracts revealed antioxidant activities similar to or higher than ascorbic acid. To our knowledge, no previous studies have been reported regarding the antioxidant or scavenging properties and a phytochemical assessment of *F. prolixa* extract.

These results highlight the potential of *F. prolixa* aerial roots as a source of antioxidants for skin care topical applications.

## Figures and Tables

**Table 1:** Ultrasound-assisted extraction yield and total phenolic content.

Plant Species	Plant Parts	Extraction Yield (%, m/m)	TPC (mg GAE/g Extract) ^1^
*Ficus prolixa*	aerial roots	16.5	148
*Calophyllum inophyllum*	leaves	21.5	143
*Curcuma longa*	rhizomes	24.6	140
*Cordia subcordata*	leaves	17.5	139
*Gardenia taitensis*	flowers	30.8	75
*Calophyllum inophyllum*	nuts	33.1	71

^1^ GAE Gallic Acid Equivalent.

**Table 2:** Yields, scavenging, and antioxidant capacity of *F. prolixa* L/L extracts.

L/L Extracts and Positive Control	Extraction Yield (%, m/m)	DPPH EC_50_ (µg/mL)	SD	FRAP (mmol Fe^2+^/g)	SD
Hexane	3.4	ND		ND	
Dichloromethane	2.7	ND		ND	
Ethyl acetate	5	3.87	±0.2	9.36	±1.2
Butanol	15.7	3.28	±0.0	9.18	±1.0
Ascorbic acid ^1^	ND	5.17	±0.1	11.67	±0.9

^1^ Positive control; ND Not Determined; SD Standard deviation.

## Data Availability

The authors ensure that this manuscript adheres to the transparency guidelines by disclosing all noteworthy aspects of the study being reported. The data presented in this study are openly available. Data are available in a publicly accessible repository. Raw LC/MS data are available at: ftp://massive.ucsd.edu/MSV000092788/ (accessed on 1 September 2023) and and treated LC/MS data at: https://doi.org/10.5281/zenodo.8300733 (accessed on 1 September 2023). GNPS Feature-Based Molecular Networking Jobs are available online at: https://gnps.ucsd.edu/ProteoSAFe/status.jsp?task=b6c3f78c95864bd7b32dc3d9c718048e (accessed on 1 September 2023) and https://gnps.ucsd.edu/ProteoSAFe/status.jsp?task=807ba58b73a34ace9b3aea96f9361e4c (accessed on 1 September 2023), for positive and negative modes, respectively.

## Supplementary Materials

The following supporting information can be downloaded at: https://www.mdpi.com/article/10.3390/antiox12101870/s1, Figure S1. On-Line RP HPLC DPPH assay chromatogram profiles of inactive extract; Figure S2. Molecular Network cluster created with GNPS using spectral data of crude extracts in negative mode; Table S1. Studied plant presentation; Table S2. MZmine parameters.
