# Supplementary material for: Identification of Antioxidant Metabolites from Five Plants (Calophyllum inophyllum, Gardenia taitensis, Curcuma longa, Cordia subcordata, Ficus prolixa) of the Polynesian Pharmacopoeia and Cosmetopoeia for Skin Care
Source: Antioxidants (Basel). 2023 Oct 16;12(10):1870. doi: 10.3390/antiox12101870 (PMC10604782; doi:10.3390/antiox12101870)
Supplement: Supplementary file 1 [file antioxidants-12-01870-s001.zip › Supplementary data/Supplementary data_Chambon et al.pdf]

Identification of antioxidant metabolites from Five Plants of the Polynesian Pharmacopoeia and Cosmetopoeia for skin care

Marion Chambon [1], Raimana Ho [1], Beatrice Baghdikian [2], Gaëtan Herbette [3], Sok-Siya Bun-Llopet [2], Elnur Garayev [2], and Phila Raharivelomanana [1,*]

[1] UMR 214 EIO, Université de Polynésie Française, IFREMER, ILM, IRD, BP 6570, F-98702, Faaa, Tahiti, French Polynesia marion.chambon@doctorant.upf.pf (M.C.); raimana.ho@upf.pf (R.H.)
[2] Aix Marseille Univ, CNRS 7263, IRD 237, Avignon Université, IMBE, 27 Blvd Jean Moulin, Service of Pharmacognosy, Faculty of Pharmacy, 13385 Marseille, France; beatrice.baghdikian@univ-amu.fr (B.B.); sok-siya.bun@univ-amu.fr (S.-S.B.-L.) elnur.garayev@univ-amu.fr (E.G.)
[3] Aix Marseille Université, CNRS, Centrale Méditerranée, FSCM, Spectropole, Service 511, Campus Saint-Jérôme, 13397 Marseille, France; gaetan.herbette@univ-amu.fr (G.H.)
* Correspondence: phila.raharivelomanana@upf.pf

Table S1. Studied plant presentation

Table S2. MZmine parameters

Figure S1. On-Line RP HPLC DPPH assay chromatogram profiles of inactive extract.

Figure S2. Molecular Network cluster created with GNPS using spectral data of crude extracts in negative mode

Table S1. Studied plant presentation

***Calophyllum inophyllum* L.**

Family: Calophyllaceae
Vernacular name: Tamanu
Biogeographic status: Polynesian introduction
Parts used: Leaves and fruits (nuts)
Botanical description: Evergreen tree of 5-12 m in height. Bark gray brown exuding pellucid resins when wounded. Broadly elliptic green leaf, shiny on both surfaces. White scented flowers. Green globose drupes, yellow when mature fruit (2.5 cm in diam).

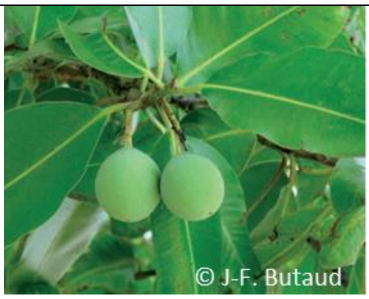

***Gardenia taitensis* DC.**

Family: Rubiaceae
Vernacular name: Tiare
Biogeographic status: Polynesian introduction
Parts used: Flowers
Botanical description: Evergreen shrub or small tree (1-6 m tall). Glossy dark green opposite leaves (5-16 cm long). Creamy white fragrant flowers, pinwheel-shaped with 5 to 9 lobes (4 cm long). Ellipsoidal fruits within numerous seeds in orange pulp at maturity.

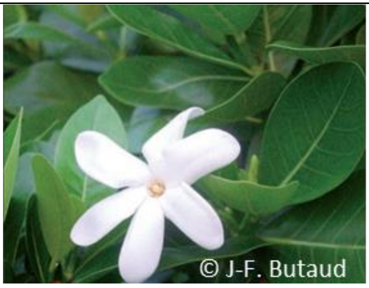

***Curcuma longa* L.**

Family: Zingiberaceae
Vernacular name: Rea
Biogeographic status: Polynesian introduction
Parts used: Rhizomes
Botanical description: Herbaceous plants of 1 m. Alternate blade green leaves (76-115 cm long). Aromatic orange-yellow cylindrical rhizomes composed by many branched. Light purple and white to green inflorescence and flowers with bright-yellow corolla tube producing small brown sterile seeds.

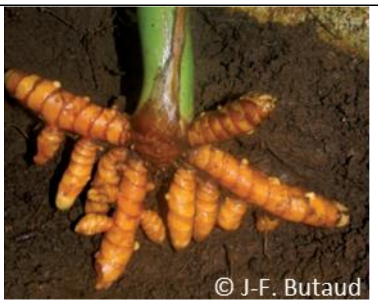

***Cordia subcordata* Lam.**

Family: Boraginaceae
Vernacular name: Tou
Biogeographic status: Indigenous
Parts used: Leaves
Botanical description: Evergreen tree of 7-15 m with yellow-brown bark, growing in coastal area. Ovate alternate leaves, light green, shiny above and dull below. Flowers with orange corolla in funnel-shaped wide at the throat (2.5-4 cm). Round fruits (2-3 cm in diam), hard and woody when mature.

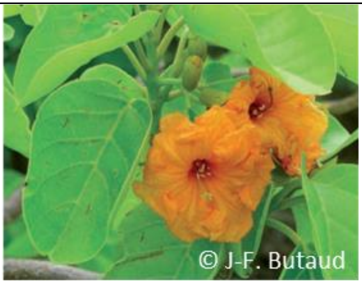

***Ficus prolixa* G.Forst.**

Family: Moraceae
Vernacular name: Ora
Biogeographic status: Indigenous
Parts used: Aerial roots
Botanical description: Large banyan tree (up to 30 m tall), much-branched. Ovate alternate dark green leaves (16 x 6.5 cm). Filiform aerial roots forming thick roots when reach the ground. Subsessile inflorescence and monoecious flowers. Little round fig white to purple when mature.

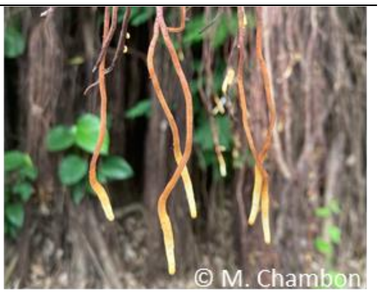

Table S2. MZmine parameters

Module	Parameters
Raw data methods<br>> Raw data import	Importation of all .mzXML files
Raw data methods<br>> Mass detection	Scans MS level : **1**<br>Mass detector : **centroid**<br>Noise level : **2.0E3**
Raw data methods<br>> Mass detection	Scans MS level : **2**<br>Mass detector : **centroid**<br>Noise level : **1.5E2**
Feature detection<br>> LC-MS<br>> ADAP chromatogram builder	Scans MS level : **1**<br>Min group group size in # of scans : **3**<br>Group intensity threshold : **2.0E3**<br>Min highest intensity : **2.0E2**<br>Scan to scan accuracy (m/z) : **10 ppm**
Feature detection > Smoothing	Smoothing algorithm: **Loess smoothing**
Feature detection<br>> Chromatogram resolving<br>> Local minimum resolver	Chromatographic threshold : **90%**<br>Minimum search range RT/Mobility: **0.10 (POS); 0.01 (NEG)**<br>Minimum relative height : **15% (POS); 1% (NEG)**<br>Minimum absolute height : **1.0E3**<br>Min ratio of peak top/edge : **1.6**<br>Peak duration range (min/mobility) : **0.01-2.3 (POS); 0.01-2.4 (NEG)**<br>Min # of data points : **3**
Feature list methods<br>> Isotopes<br>> 13C isotope filter	m/z tolerance : **10 ppm**<br>Retention time tolerance : **0.1 absolute (min)**<br>Mobility tolerance : **unchecked**<br>Monotonic shape : **unchecked**<br>Maximum charge : **2**<br>Representative isotope : **most intense**<br>Never remove feature with MS2 : **checked**
Feature list methods<br>> Alignment<br>> Join aligner	m/z tolerance : **10 ppm**<br>Weight for m/z : **75**<br>Retention time tolerance : **0.2 absolute (min)**<br>Weight for RT : **25**<br>Mobility tolerance : **unchecked**<br>Mobility weight : **1.00**<br>Require same charge state : **checked**<br>Require same ID : **unchecked**<br>Compare isotope pattern : **unchecked**<br>Compare spectra similarity : **unchecked**
Feature list methods<br>> Processing<br>> Assign MS^2 to features	Retention time tolerance : **0.1 absolute (min)**<br>MS1 to MS2 precursor tolerance (m/z) : **10 ppm**<br>Limit by RT edges : **unchecked**<br>Combine MS/MS spectra (TIMS) : **unchecked**<br>Lock to feature mobility range : **unchecked**<br>Minimum merged intensity : **unchecked**
Feature list methods<br>> Feature list filtering<br>> Feature list rows filter	Retention time : **0.6 - 30.0 min (auto range)**<br>Features with MS2 scans : **checked**
Feature list methods<br>> Feature list filtering<br>> Feature list blank subtraction	Minimum # of detection in blanks : **1**<br>Fold change increase : **unchecked**

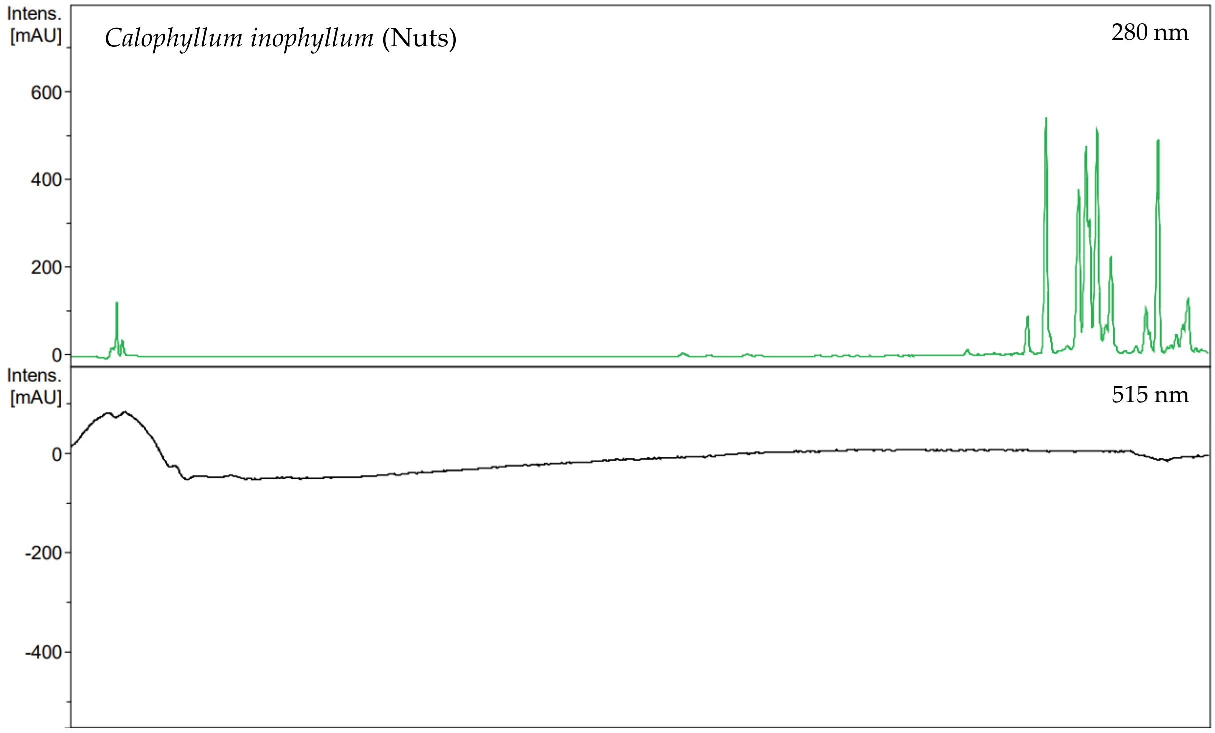

Figure S1. On-Line RP HPLC DPPH assay chromatogram profiles of inactive extract.

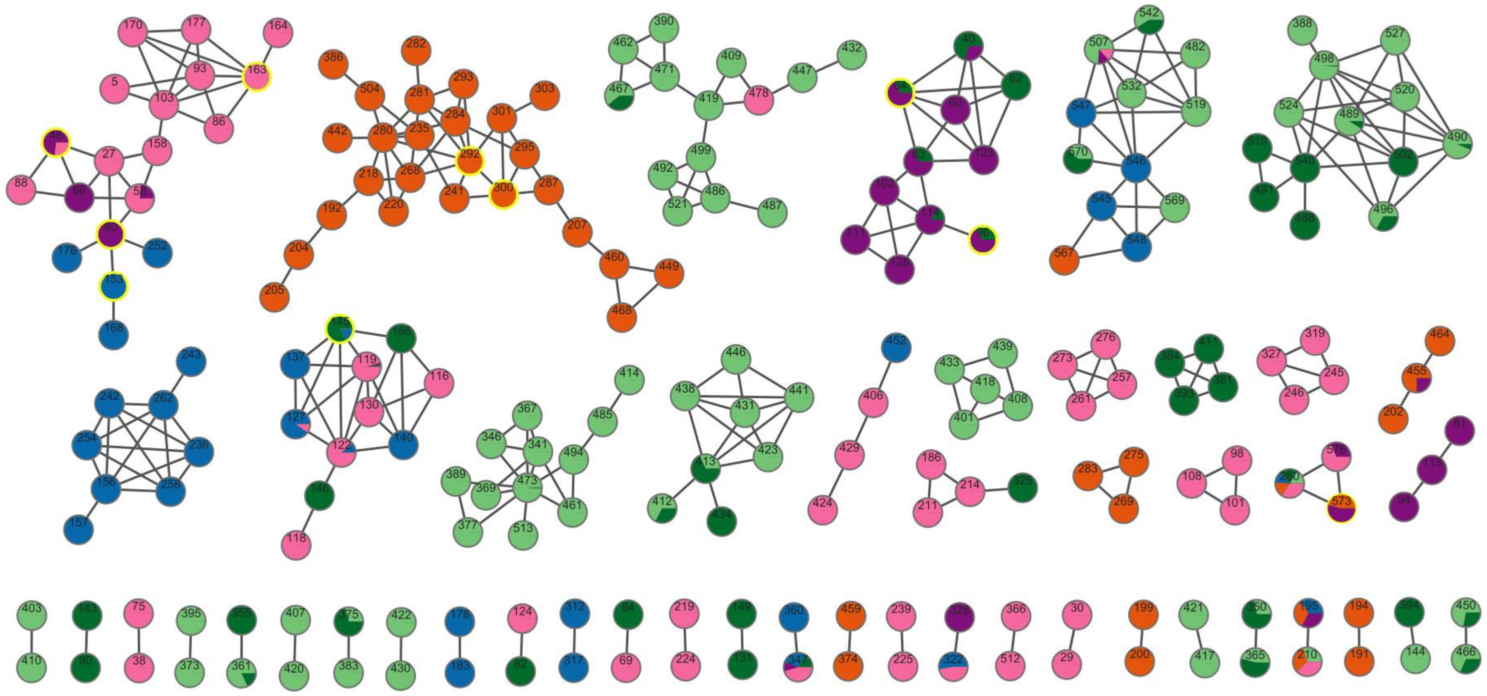

Figure S2. Molecular Network cluster created with GNPS using spectral data of crude extracts in negative mode (self-loop nodes removed).

Node colors represent repartition in plant extracts: *C. inophyllum* leaves (dark green), *C. inophyllum* nuts (light green), *F. prolixa* (purple), *C. subcordata* (blue), *G. taitensis* (pink), *C. longa* (orange).
