# Supplementary figures and images for: Identification of Antioxidant Metabolites from Five Plants (Calophyllum inophyllum, Gardenia taitensis, Curcuma longa, Cordia subcordata, Ficus prolixa) of the Polynesian Pharmacopoeia and Cosmetopoeia for Skin Care
Source: Antioxidants (Basel). 2023 Oct 16;12(10):1870. doi: 10.3390/antiox12101870 (PMC10604782; doi:10.3390/antiox12101870)

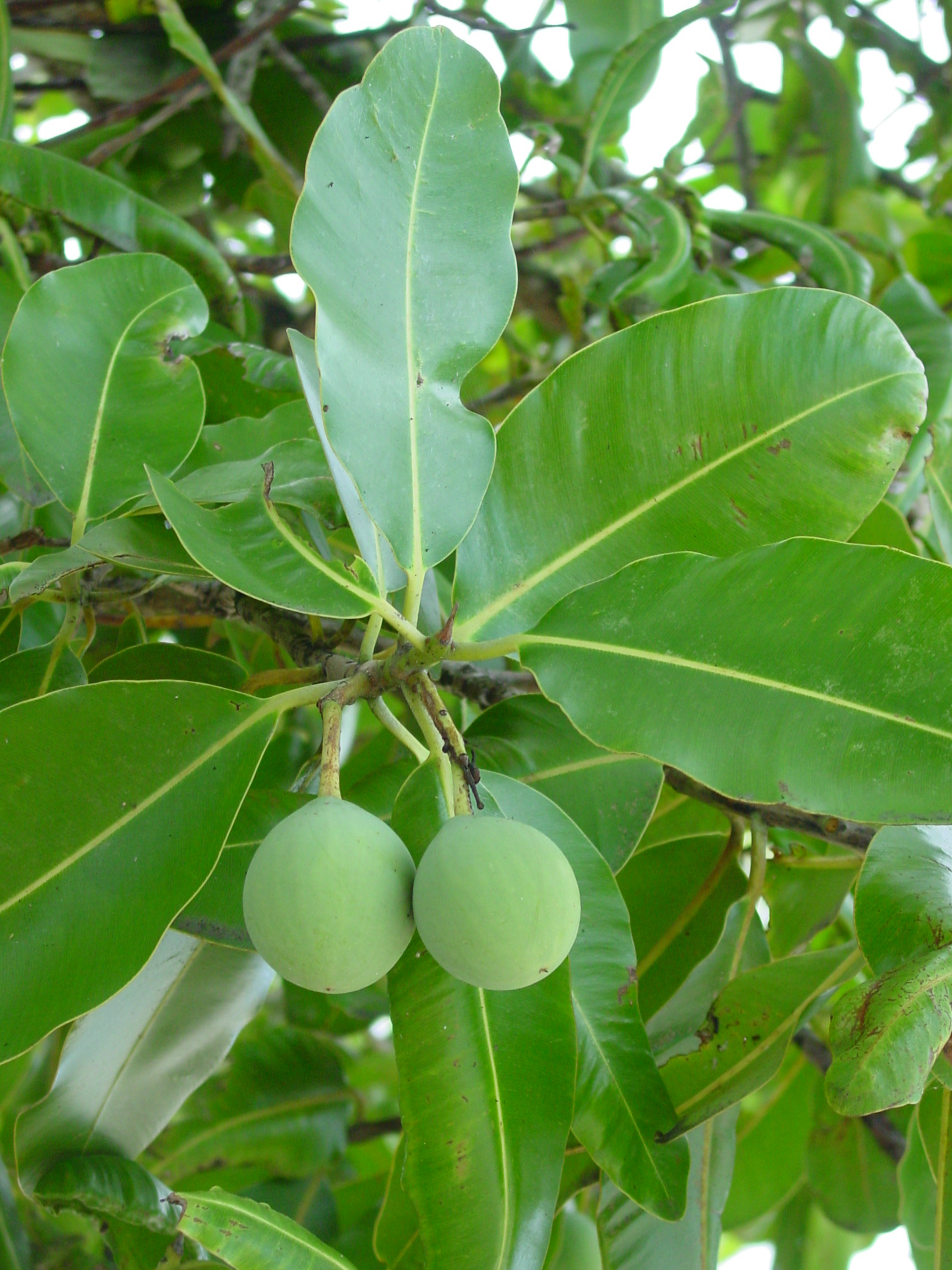

Supplement: Supplementary file 1 [file antioxidants-12-01870-s001.zip › Supplementary data/Files for figure (Supp. data)_Chambon et al/Calophyllum inophyllum.pdf]

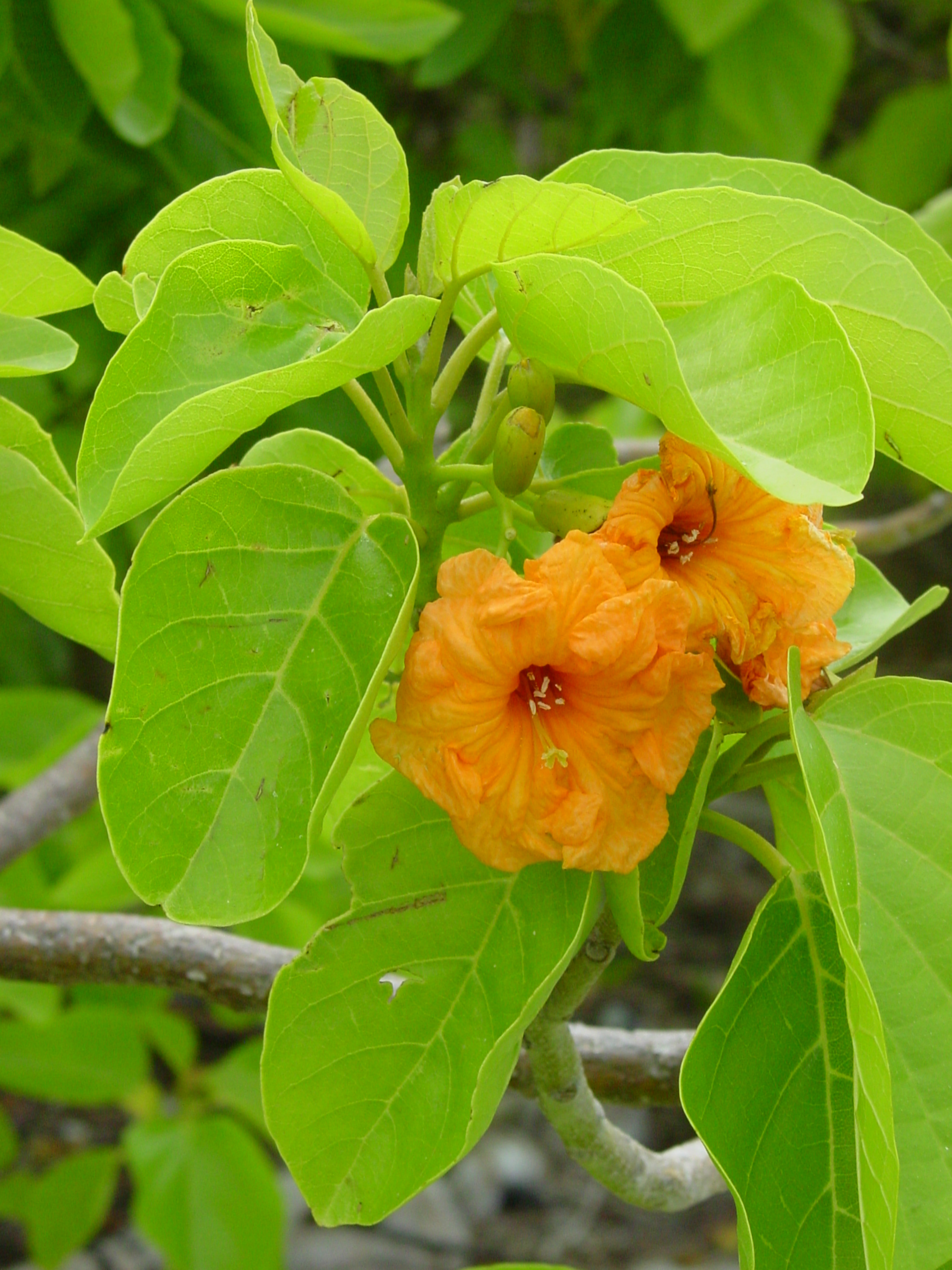

Supplement: Supplementary file 1 [file antioxidants-12-01870-s001.zip › Supplementary data/Files for figure (Supp. data)_Chambon et al/Cordia subcordata.pdf]

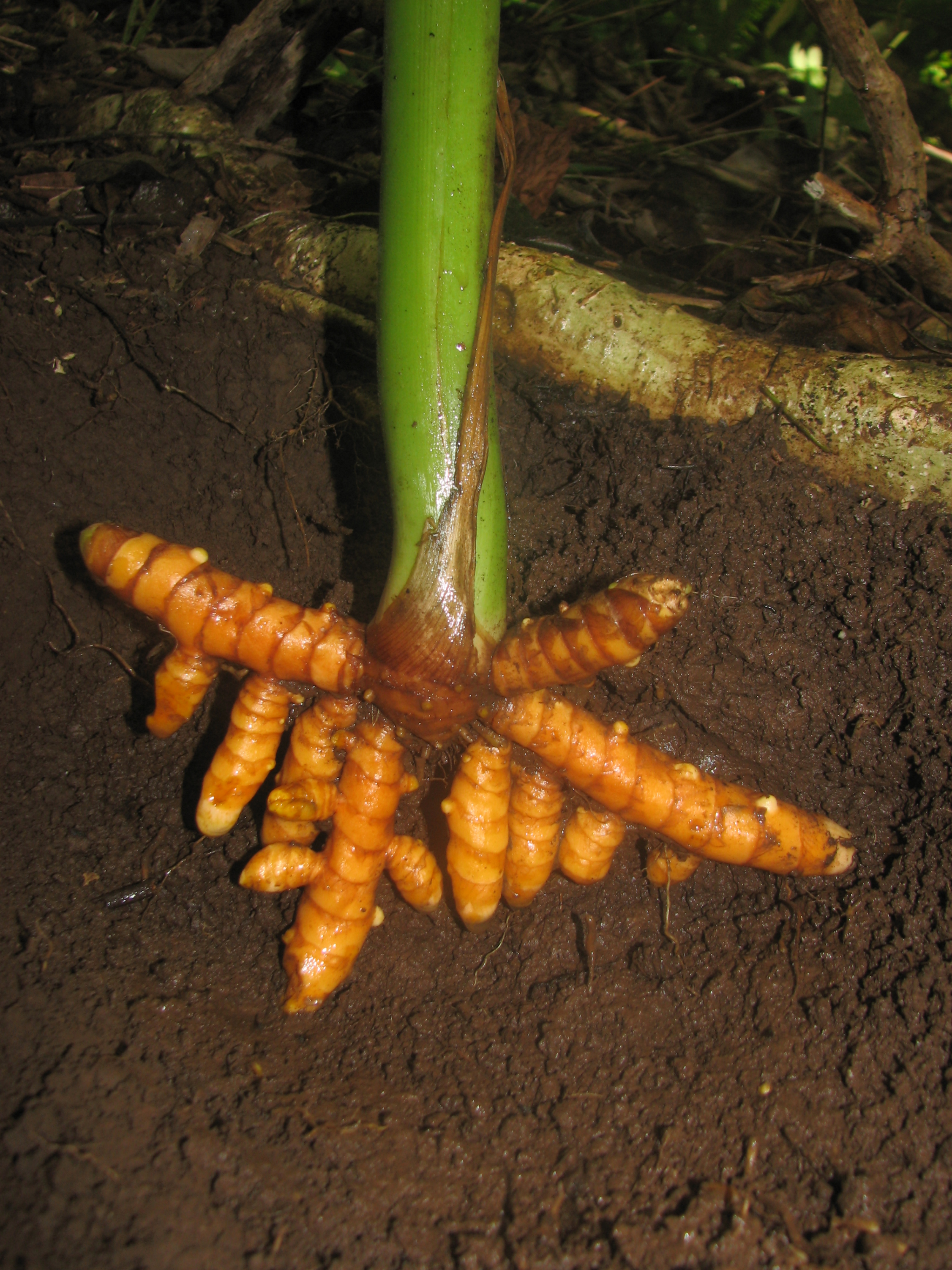

Supplement: Supplementary file 1 [file antioxidants-12-01870-s001.zip › Supplementary data/Files for figure (Supp. data)_Chambon et al/Curcuma longa.pdf]

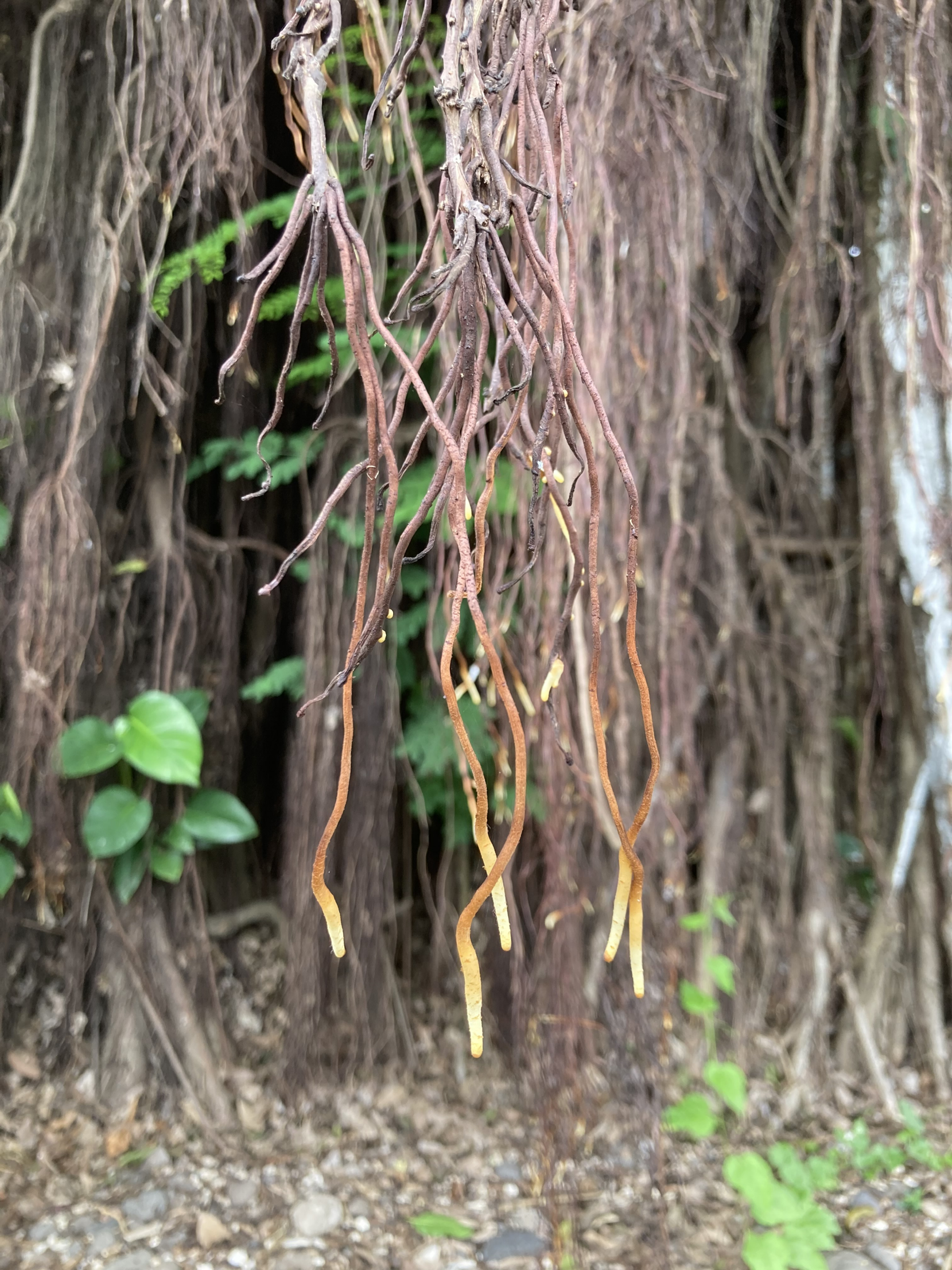

Supplement: Supplementary file 1 [file antioxidants-12-01870-s001.zip › Supplementary data/Files for figure (Supp. data)_Chambon et al/Ficus prolixa.pdf]

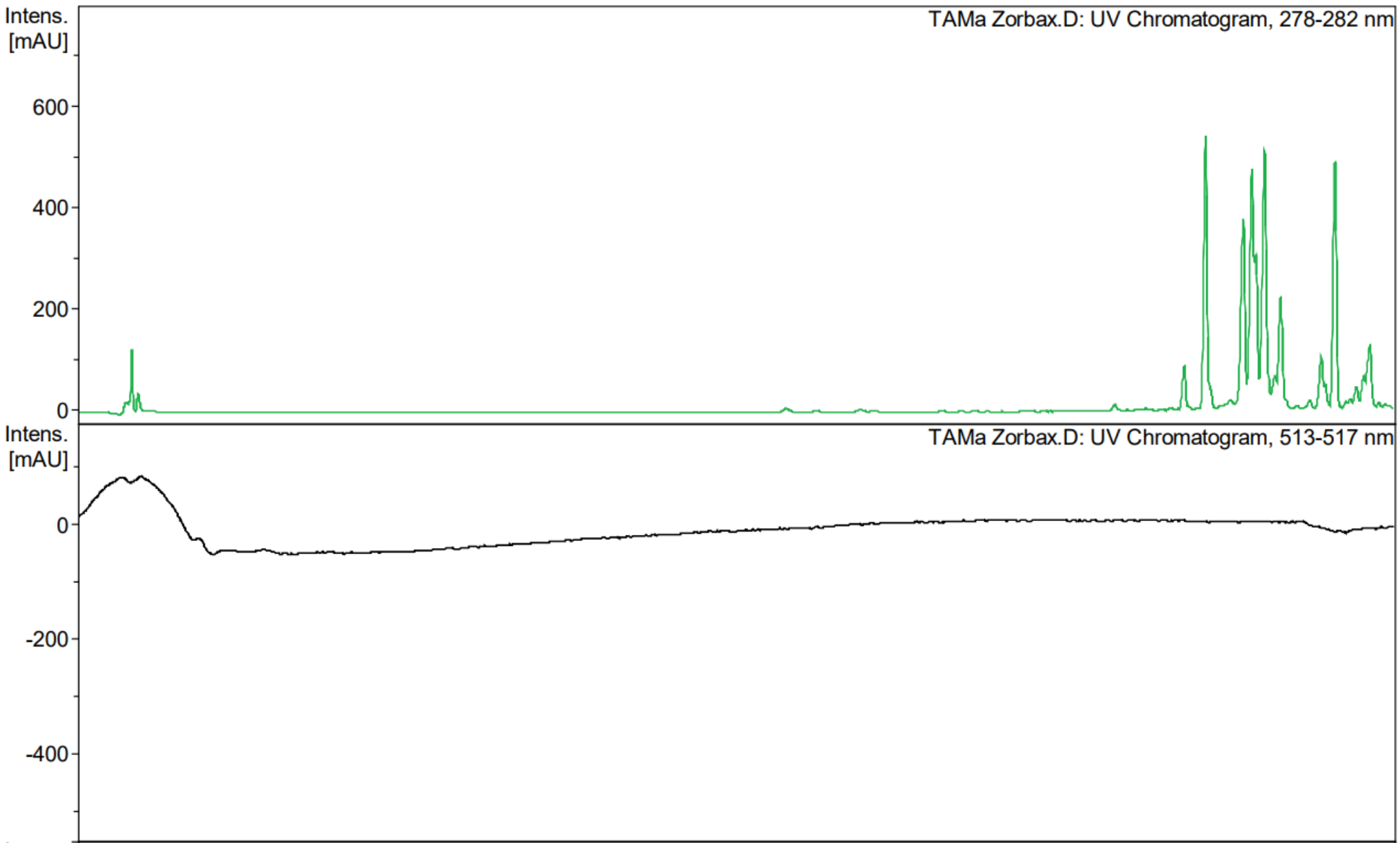

Supplement: Supplementary file 1 [file antioxidants-12-01870-s001.zip › Supplementary data/Files for figure (Supp. data)_Chambon et al/Figure S1.pdf]

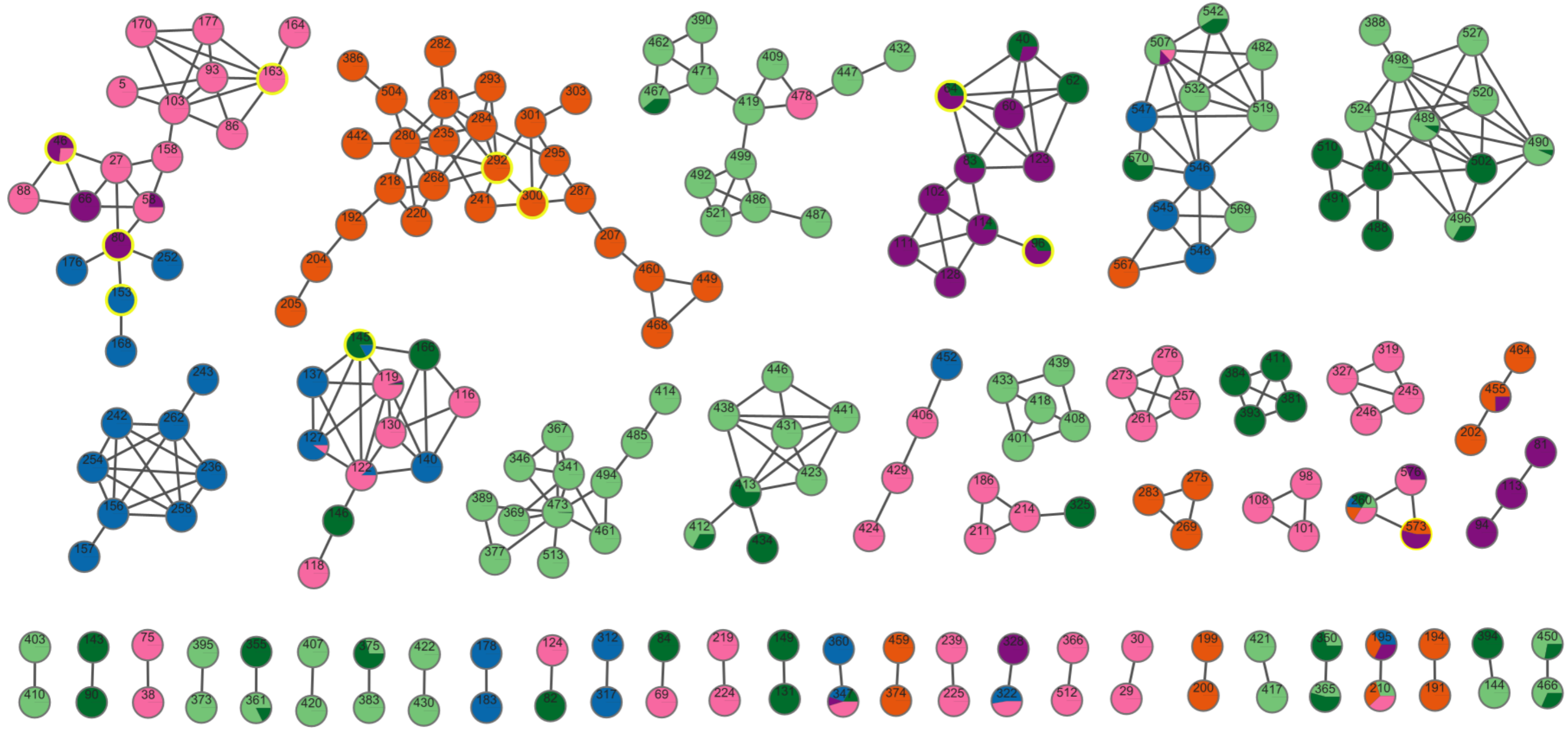

Supplement: Supplementary file 1 [file antioxidants-12-01870-s001.zip › Supplementary data/Files for figure (Supp. data)_Chambon et al/Figure S2.pdf]

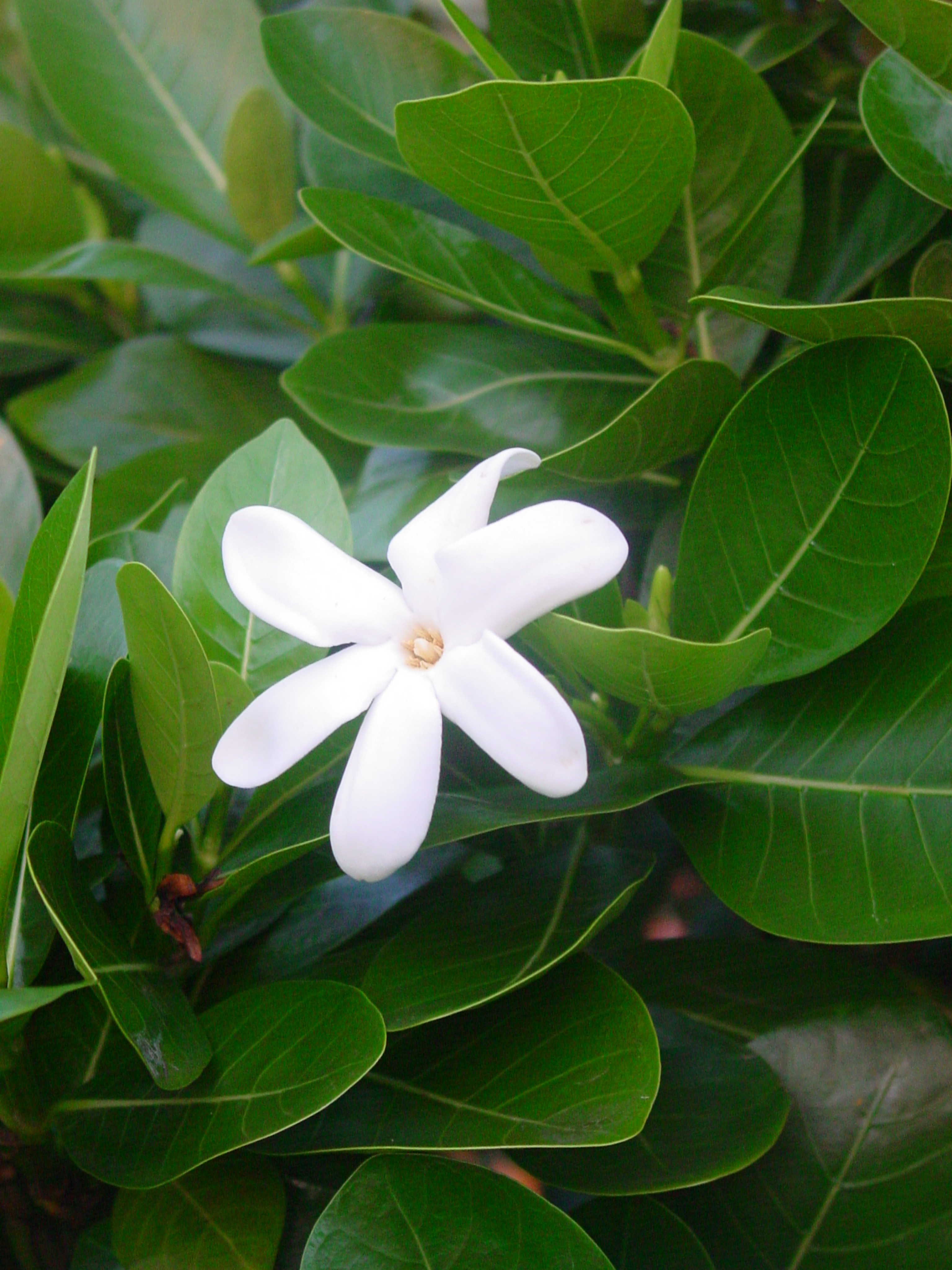

Supplement: Supplementary file 1 [file antioxidants-12-01870-s001.zip › Supplementary data/Files for figure (Supp. data)_Chambon et al/Gardenia taitensis.pdf]
